# Efficacy and safety of vonoprazan versus proton pump inhibitors in the treatment of peptic ulcer disease: a systematic review and network meta-analysis for randomized controlled trails

**DOI:** 10.3389/fnut.2024.1436993

**Published:** 2024-09-05

**Authors:** Lidi Tian, Dan Xiang, Feili Yue, Runjie Li, Youping Zhou

**Affiliations:** ^1^Department of Dermatology, Ya’an People’s Hospital, Ya’an, China; ^2^Department of Clinical Medicine, Ya’an People’s Hospital, Ya’an, China; ^3^Department of Geriatric Medicine, Ya’an People’s Hospital, Ya’an, China; ^4^Department of Gastroenterology, The First Affiliated Hospital of Chongqing Medical University, Chongqing, China

**Keywords:** peptic ulcer disease, vonoprazan, proton pump inhibitors, safety, efficacy

## Abstract

**Background and aims:**

Vonoprazan, a novel acid suppressant, has been employed in the treatment of peptic ulcer disease in recent years. However, the efficacy and safety of vonoprazan versus proton-pump inhibitors remains controversial. To address this gap, a systematic review and network meta-analysis were conducted to evaluate the efficacy and safety of vonoprazan in comparison with various proton-pump inhibitors.

**Methods:**

Randomized controlled trials that met selection criteria in PubMed (Medline), EMBASE and the Cochrane Library were searched up to July 15, 2024. The primary outcome was ulcer healing rate. Secondary outcomes were treatment-emergent adverse events and drug-related adverse events. Effect size on outcomes is presented as odds ratios with 95% confidence intervals.

**Results:**

Thirty-five randomized controlled trials containing 9,544 participants were included. In terms of the healing rate at 2 weeks, lansoprazole 30 mg ranked first, followed by vonoprazan 20 mg and ilaprazole 10 mg. In terms of the healing rate at 4 weeks, pantoprazole 40 mg ranked first, with rabeprazole 10 mg and lansoprazole 30 mg ranking second and third, respectively. Regarding the healing rate at 8 weeks, lansoprazole 30 mg is demonstrated to be the most efficacious regimen. Moreover, subgroup analysis indicated that lansoprazole 30 mg is the optimal regimen in the treatment of artificial gastric ulcer at 4 and 8 weeks. Importantly, lansoprazole 30 mg has fewer adverse reactions and higher safety.

**Conclusion:**

The optimal regimen for the treatment of peptic ulcer disease may be lansoprazole 30 mg at 2 and 8 weeks, while pantoprazole 40 mg has demonstrated superior performance at the 4-week when compared to vonoprazan 20 mg. Furthermore, lansoprazole 30 mg has shown to be superior in terms of safety outcomes. These findings, derived from a network meta-analysis, necessitate further research for validation.

## Introduction

Peptic ulcer disease (PUD), a common gastrointestinal disorder, is usually defined as gastric or duodenal injury, resulting in mucosal rupture reaching the submucosa (1, 2). The two main risk factors for gastrointestinal injury and acid-related peptic ulcers are *Helicobacter pylori* (*H. pylori*) infection and the use of nonsteroidal anti-inflammatory drugs (NSAIDs) (1, 2).The estimated lifetime prevalence of PUD is 5–10% in the general population, and the annual incidence rate is 0.1–0.3% (2, 3). Although morbidity and mortality due to PUD have decreased significantly from 1990 to 2019, the fatality rate remains high (4, 5). Nowadays, the treatment strategy for PUD is to reduce the damage to gastrointestinal mucosa and promote ulcer healing by inhibiting gastric acid secretion. Proton pump inhibitors (PPIs) constitute a class of acid-suppressing pharmacological agents frequently employed in the management of PUD. These agents function by irreversibly inhibiting the proton pump (H+/K+/ATPase), thereby reducing gastric acid secretion (6, 7). The advent of PPIs has markedly transformed the therapeutic approach to PUD, significantly enhancing its healing rates. However, despite their status as the most efficacious treatment for PUD, PPIs exhibit certain limitations. These include a short half-life, the necessity for acid activation, a relatively slow onset of clinical effect, and variability in clinical response due to polymorphisms in the CYP2C19 enzyme (7–10).

Vonoprazan, a novel potassium-competitive acid blocker (P-CAB), presents a promising alternative to PPIs. It exerts its effect by reversibly inhibiting gastric acid secretion through competitive blockade of K+ binding to gastric H+/K+ ATPase (11). Studies have demonstrated that vonoprazan achieves more rapid and potent acid suppression, reaching maximum plasma concentration within 2 h, significantly faster than PPIs (11, 12). Additionally, vonoprazan demonstrates a slower dissociation rate from the proton pump, leading to an extended duration of acid inhibition with a plasma half-life of up to 9 h (11, 12). Furthermore, the plasma concentration and anti-secretory effects of vonoprazan are notably stable, and its efficacy remains unaffected by dietary factors and genetic polymorphisms, which significantly impact the performance of most PPIs (12, 13).

Recently, a substantial body of research has examined the efficacy and safety of vonoprazan and PPIs in the treatment of PUD, with results exhibiting considerable variability (14–22). Some of the studies (14, 16–18, 22) verified both non-inferiority and equivalence of vonoprazan 20 mg to lansoprazole 30 mg, one of the most commonly used PPIs, for the treatment of PUD. Other studies (15, 20) demonstrated both non-inferiority and superiority of vonoprazan 20 mg to esomeprazole 20 mg in the ulcer healing of endoscopic submucosal dissection (ESD) induced gastric ulcer. Komori et al. (21) pointed out that vonoprazan 20 mg was not superior to rebeprazole 10 mg in ESD-induced gastric ulcer. However, there is a paucity of research comparing the efficacy of vonoprazan and PPIs other than lansoprazole, esomeprazole and rebeprazole in the management of PUD. To address this gap, we conducted a network meta-analysis of randomized controlled trials (RCTs) to evaluate the efficacy and safety of different doses of vonoprazan relative to different doses of PPIs in the treatment of PUD.

## Methods

This network meta-analysis was performed using the Preferred Reporting Items for Systematic Reviews and Meta-analysis (PRISMA) statement (23), and the PRISMA checklist was shown in Supplementary Table S1. The study protocol was registered on PROSPERO with registration number CRD42023442859.

### Search strategy

From inception to July 15, 2024, potentially relevant studies published that examined the efficacy or safety of PPIs with vonoprazan or placebo in PUD were searched using the following electronic databases: PubMed (Medline), EMBASE and the Cochrane Library. The search terms included the following keywords: peptic ulcer, gastric ulcer, duodenal ulcer, PCABs, Vonoprazan, PPIs and RCTs. Reference lists of the relevant studies were also hand-searched for potentially related studies. The search strategy was shown in Supplementary Table S2.

### Study selection

Potentially relevant published studies underwent a review of the entire published manuscript by two independent researchers (FY and RL). The inclusion criteria were (a) adult patients who underwent PUD, including gastric ulcer or duodenal ulcer; (b) interventions including ilaprazole, omeprazole, esomeprazole, pantoprazole, lansoprazole, dexlansoprazole, rabeprazole, anaprazole and vonoprazan with different doses; (c) the control group could be a placebo or a comparison between above drugs; (d) outcomes: ulcer healing rates for treatment durations between 2 and 8 weeks, rates of treatment-emergent adverse events (TEAEs), and drug-related adverse events (DRAEs); (e) study design: RCTs. The exclusion criteria were (a) publications in languages other than English, (b) lack of necessary outcomes to be extracted, (c) treatment with vonoprazan or PPIs mentioned above in combination with other medications. Any disagreement was resolved by discussion with a third researcher (DX).

### Data extraction and study quality assessment

Two reviewers (FY and RL) independently extracted the following information from each included study: first author; year of publication; country; study period; details of each treatment regimen; sample size; follow-up duration; clinical outcomes, including efficacy outcomes and safety outcomes. Efficacy analysis was based on the ulcer healing rate of PUD, as confirmed by the endoscopy; safety analysis was based on TEAEs and DRAEs. TEAEs: Adverse events that arise after the initiation of treatment and include any new or worsening conditions during the treatment period. DRAEs: A subset of adverse events that are directly attributed to the drug treatment, based on clinical judgment.

Per-protocol (PP) data were collected for efficacy outcome when possible; otherwise, intention-to-treat (ITT) data were collected. For safety outcomes, only ITT data were collected and included. Disagreement was resolved after discussion with another researcher (DX). The Cochrane Risk of Bias assessment tool was used for assessing the risk of bias in individual studies (24).

### Statistical analysis

All statistical analyses in the current meta-analysis were performed to calculate the direct and indirect evidence on efficacy and safety of different treatments based on the frequentist framework. Binary variables were calculated using odds ratio (OR) with 95% confidence intervals (CIs). The inconsistency assessment comprised global inconsistency and local inconsistency (25). Global inconsistency was estimated by a design-by-treatment interaction model, and local inconsistency was estimated by the node-splitting method. *p* > 0.05 suggested no significant inconsistency, and we would conduct network meta-analysis with a random-effects model. We calculated the surface under the cumulative ranking (SUCRA) to estimate the cumulative ranking for each treatment. SUCRA is used to provide a summary measure of the effectiveness of each treatment within the network meta-analysis framework. It represents the probability that a treatment is among the best options available. SUCRA values range from 0 to 1, where higher values indicate a higher likelihood of a treatment being among the top-ranked treatments (26). In addition, we also assessed potential small-trial effects and publication bias for all available comparisons with sufficient studies (≥10 studies), and *p* < 0.05 indicated the existence of publication bias (27). All data analyses were conducted through STATA 16.0 and Review Manager version 5.3.

## Results

### Literature selection

**Figure 1:**
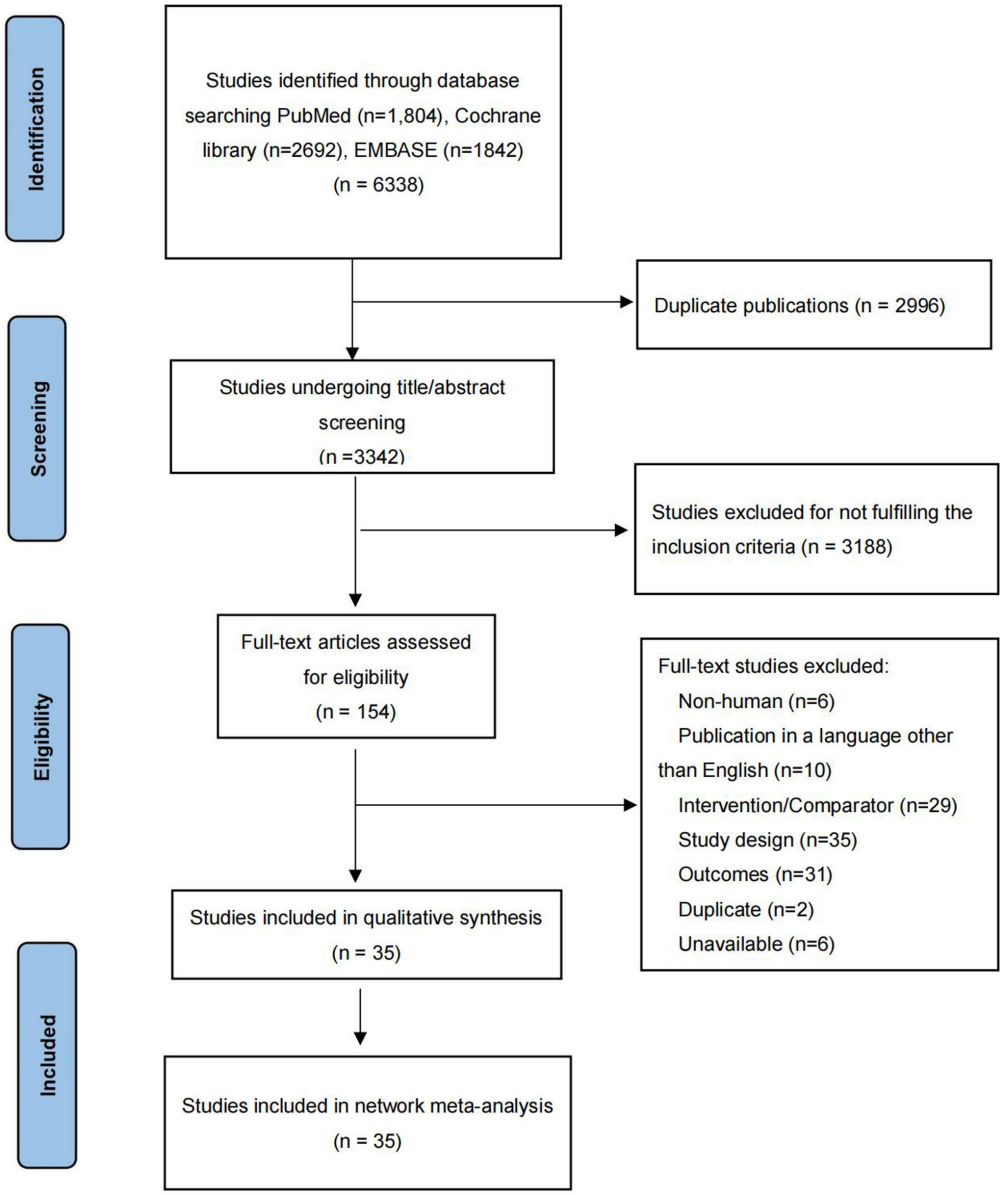
Flowchart of literature selection.

The initial literature search identified 6,338 potentially relevant articles. After removing 2,996 duplicates, the titles and abstracts of remaining 3,342 articles were primary screened. 154 potentially eligible articles were further estimated by full-text review. As shown in Figure 1, 35 RCTs including a total of 9,544 participants were finally included (14–22, 28–53).

### Based characteristics of eligible studies and quality assessment

The included studies investigated seven different drugs, including omeprazole, lansoprazole, pantoprazole, ilaprazole, rabeprazole, esomeprazole and vonoprazan. All the included studies were published from 1990 to 2022. The sample sizes of the included studies ranged from 12 to 817. One of the included studies (43) had four arms, two of the studies (42, 52) had three arms, while the rest of the studies all had two arms (14–22, 28–41, 44–51, 53). Eleven studies (28, 29, 31–33, 35–39, 46) were conducted in European countries or USA, twenty-four studies (14–22, 30, 34, 40–45, 47–53) were conducted in Asia. Among the included studies, eight studies (28, 29, 34, 35, 39, 44, 46, 47) were placebo-controlled trials, the remaining twenty-seven studies (30–33, 36–38, 40–43, 45, 48–53) compared different regimens of drugs. The main characteristics of included studies are summary in Table 1.

**Table 1:** Basic characteristics of included studies.

First author	Year	Country	Treatment		Number of patients		Age(year); mean/media		Study period	Follow-up duration	Outcome measure	
			Group A	Group B	Group A	Group B	Group A	Group B			Efficacy	Safety
Graham (28)	1990	USA	OPZ 20 mg qd	Placebo	102	51	47.5	51	/	4 weeks	√	√
Avner (29)	1995	USA	LPZ 30 mg qd	Placebo	75	75	41.3	44.7	/	4 weeks	√	√
Chang (30)	1995	China	LPZ 30 mg qd	OPZ 20 mg qd	57	54	56.4	59.3	/	4 weeks	√	√
Ekström (31)	1995	Sweden	LPZ 30 mg qd	OPZ 20 mg qd	143	136	54.4	55.3	1990.2–1991.8	4 weeks	√	√
Witzel (32)	1995	Germany	PPZ 40 mg qd	OPZ 20 mg qd	163	80	56	57	/	8 weeks	√	√
Rehner (33)	1995	Germany	PPZ 40 mg qd	OPZ 20 mg qd	193	93	46	47	/	4 weeks	√	√
Goh (34)	1995	China	OPZ 20 mg qd	Placebo	60	63	45	48	/	12 months		√
Kovacs (35)	1998	USA	LPZ 30 mg qd	Placebo	15	15	57.5	58.3	1990.7–1991.11	12 months		√
Dekkers (36)	1998	UK	RPZ 20 mg qd	OPZ 20 mg qd	113	114	55.5	55.21	/	6 weeks	√	
Dobrilla (37)	1999	Italy	LPZ 30 mg qd	OPZ 20 mg qd	71	73	/	/	1993.3–1994.2	12 months		√
Dekkers (38)	1999	UK	RPZ 20 mg qd	OPZ 20 mg qd	102	103	47.3	47.8	/	4 weeks	√	
Bianchi (39)	2000	Italy	PPZ 40 mg qd	Placebo	70	34	58	59	/	12 weeks		√
Ando (40)	2005	Japan	RPZ 10 mg qd	OPZ 20 mg qd	39	41	51.6	50.6	2002.7–2003.10	8 weeks		√
Ji (41)	2005	South Korea	RPZ 10 mg qd	OPZ 20 mg qd	56	56	49.4	51.9	2002.7–2003.10	6 weeks	√	√
Ho (42)	2009	Singapore	IPZ 5 mg qd	OPZ 20 mg qd	74	75	52.75	52.92	2002.9–2004.2	4 weeks	√	√
			IPZ 10 mg qd		73		54.28	52.92				
			IPZ 5 mg qd	OPZ 20 mg qd	106	110	49.63	51.56				
			IPZ 10 mg qd		101		47.93					
Wang (43)	2011	China	IPZ 5 mg qd	OPZ 20 mg qd	59	59	39.3	39.2	2004.11–2005.1	4 weeks	√	√
			IPZ 10 mg qd		58		39.0		2004.11–2005.1			
			IPZ 20 mg qd		59		40.0		2004.11–2005.1			
Sugano (44)	2012	Japan	EPZ 20 mg qd	Placebo	175	168	63.6	62.4	2007.8–2009.2	24 weeks		√
Wang (45)	2012	China	IPZ 10 mg qd	OPZ 20 mg qd	331	165	41.21	40.89	2005.10–2006.1	4 weeks	√	√
Scheiman (46)	2011	USA	EPZ 20 mg qd	Placebo	804	805	67.7	67.4	2007.2–2008.8	26 weeks		√
Sugano (47)	2014	Japan	EPZ 20 mg qd	Placebo	182	182	66.1	68.1	2010.2–2012.1	72 weeks		√
Miwa (14)	2016	Japan	VPZ 20 mg qd	LPZ 30 mg qd	244	238	58.2	58.6	2011.11–2012.11	8 weeks	√	√
			VPZ 20 mg qd	LPZ 30 mg qd	184	188	49.9	50.2	2011.10–2013.2	6 weeks	√	√
Takahashi (48)	2016	Japan	VPZ 20 mg qd	LPZ 30 mg qd	14	12	71.9	74.8	2015.8–2016.3	4 weeks	√	
Tsuchiya (15)	2017	Japan	VPZ 20 mg qd	EPZ 20 mg qd	39	41	73	74	2015.4–2016.6	8 weeks	√	√
Mizokami (49)	2017	Japan	VPZ 20 mg qd	VPZ 10 mg qd	212	218	64.9	65	2011.10–2013-7	24 weeks		√
Kawai (50)	2017	Japan	VPZ 20 mg qd	VPZ 10 mg qd	202	202	69.1	68.9	2011.10–2013.4	24 weeks		√
Hirai (18)	2018	Japan	VPZ 20 mg qd	LPZ 30 mg qd	74	75	73.16	69.93	2015.4–2017.5	8 weeks	√	√
Ishii (19)	2018	Japan	VPZ 20 mg qd	EPZ 20 mg qd	27	26	70.2	70	2015.5–2017.5	8 weeks	√	√
Bang (51)	2018	South Korea	IPZ 20 mg qd	RPZ 20 mg qd	78	79	62	64	2015.6–2018.3	8 weeks	√	√
Fan (52)	2019	China	IPZ 10 mg qd	RPZ 10 mg qd	130	129	37.64	39.25	2008.11–2010.1	4 weeks	√	√
			IPZ 5 mg qd		131		40.75					
Hamada (53)	2018	Japan	VPZ 20 mg qd	LPZ 30 mg qd	69	70	70.3	70.1	/	8 weeks	√	√
Ichida (20)	2018	Japan	VPZ 20 mg qd	EPZ 20 mg qd	43	39	72.4	73.9	2015.9–2017.11	8 weeks	√	√
Komori (21)	2019	Japan	VPZ 20 mg qd	RPZ 10 mg qd	18	15	69	70.9	2015.4–2016.1	4 weeks	√	√
Ban (22)	2021	Japan	VPZ 20 mg qd	LPZ 30 mg qd	101	95	71.5	71.2	2015.9–2018.8	8 weeks	√	√
Kawai (16)	2021	Japan	VPZ 20 mg qd	LPZ 30 mg qd	85	83	73	73	2015.4–2017.11	8 weeks	√	√
Hou (17)	2022	China	VPZ 20 mg qd	LPZ 30 mg qd	265	268	42.0	41.4	2017.4–2019.7	6 weeks	√	√

VPZ, vonoprazan; OPZ, omeprazole; RPZ, rebeprazole; LPZ, lansoprazole; EPZ, esomeprazole; IPZ, Ilaprazole; PPZ, pantoprazole. “/” means no data provided and “√” indicates which specific outcome measure was assessed.

We conducted a comprehensive assessment of the risk of bias for each included study using the Cochrane risk of bias tool. The results are summarized as follows and detailed in Supplementary Figure S1. Out of the 35 studies included, 22 studies (14–17, 19–21, 31, 34, 39, 42–53) demonstrated a low risk of bias in random sequence generation by employing adequate methods. Only eighteen studies (14–17, 20, 31, 34, 39, 42–50, 52) used appropriate allocation concealment. Twenty-seven studies (14, 15, 17–19, 28–40, 42–47, 49, 50, 52) had a low bias of blinding of participants and personnel, ensuring that neither the participants nor the researchers knew which treatment was being administered. Twenty-eight studies (14, 15, 17–19, 28–47, 49, 50, 52) had a low risk of bias for blinding of outcome assessment, indicating that the outcome assessors were blinded to the treatment groups. Thirty-three studies (14–20, 22, 28–46, 48–53) addressed incomplete outcome data adequately by providing comprehensive follow-up data and accounting for all participants initially enrolled in the trials. Ten studies (14, 17, 44–47, 49–51) were identified to have other potential sources of bias, primarily related to funding or sponsorship from pharmaceutical companies.

### Efficacy outcomes

#### Ulcer healing rate at 2 weeks

**Figure 2:**
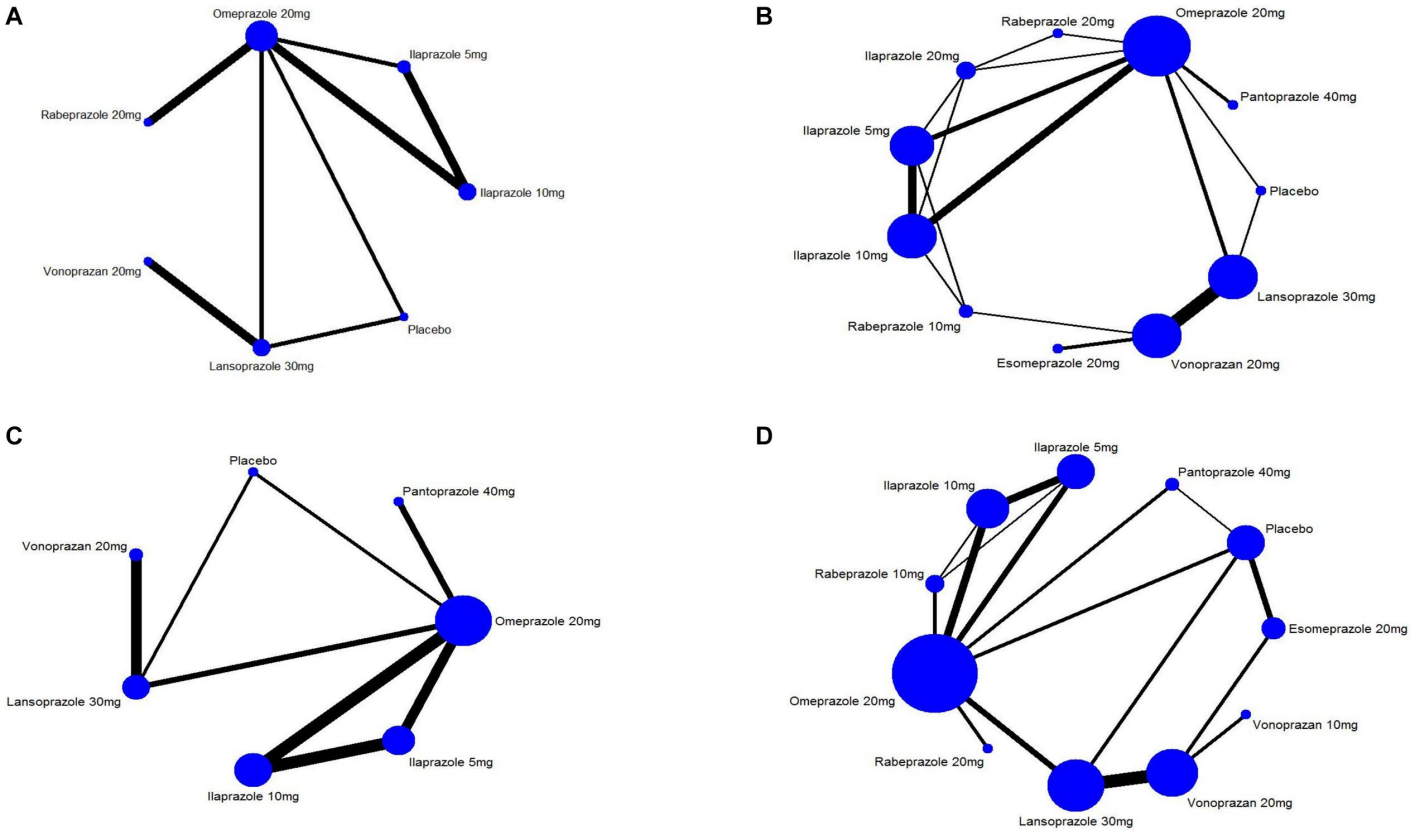
Network graph of included trails for **(A)** 2 weeks ulcer healing rates of peptic ulcer disease, **(B)** 4 weeks of ulcer healing rates of peptic ulcer disease, **(C)** 4 weeks ulcer healing rate of peptic ulcer and **(D)** treatment-emergent adverse events.

**Figure 3:**
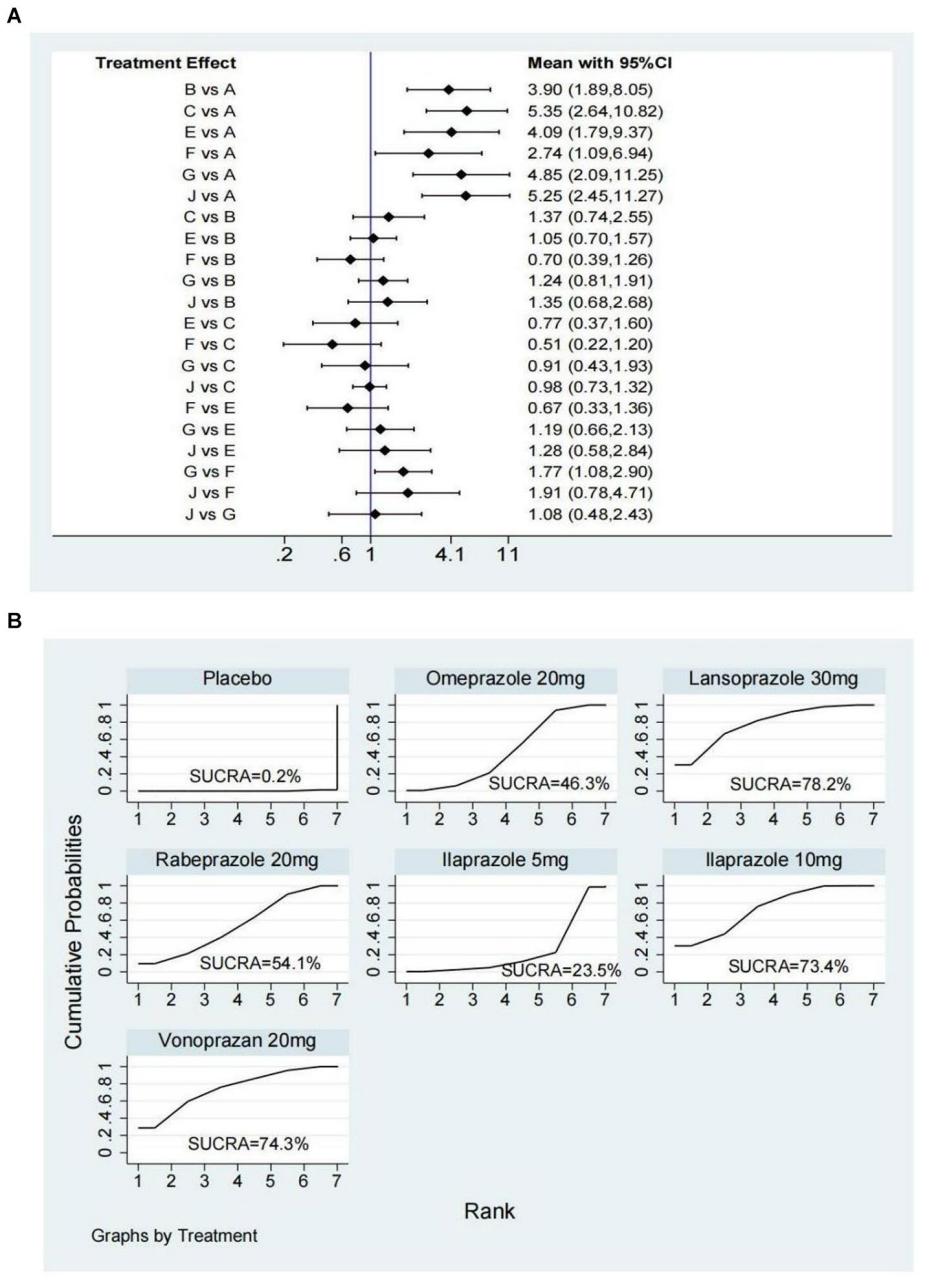
Forest plot **(A)** and surface under the cumulative ranking score (SUCRA) **(B)** of network meta-analysis for the 2-week ulcer healing rate in patients with peptic ulcer disease. A, Placebo; B, Omeprazole 20 mg; C, Lansoprazole 30 mg; E, Rabeprazole 20 mg; F, Ilaprazole 5 mg; G, Ilaprazole 10 mg; J, Vonoprazan 20 mg.

Nine studies (14, 28, 29, 31, 36, 38, 43, 45, 52) have reported the ulcer healing rate at 2 weeks, and six interventions were involved, including omeprazole 20 mg, ilaprazole 5 mg, ilaprazole 10 mg, lansoprazole 30 mg, vonoprazan 20 mg, and rabeprazole 20 mg. The network plot was shown in Figure 2A. Each node represents a drug, and the line between nodes reflects direct comparison. The sizes of nodes and widths of lines are proportional to participant numbers and trial numbers, respectively. The results of this study indicate that, compared with placebo, all interventions significantly improved the 2-week ulcer healing rate. Additionally, ilaprazole 10 mg is superior to ilaprazole 5 mg in improving the ulcer healing rate (OR = 1.77, 95%CI = 1.08–2.90) (Figure 3A).

The SUCRA ranks of efficacy of all investigated drugs are shown in Table 1 and Figure 3B. Lansoprazole 30 mg ranks first, sequentially followed by vonoprazan 20 mg, ilaprazole 10 mg, rabeprazole 20 mg, omeprazole 20 mg, ilaprazole 5 mg, and placebo.

#### Ulcer healing rate at 4 weeks

**Figure 4:**
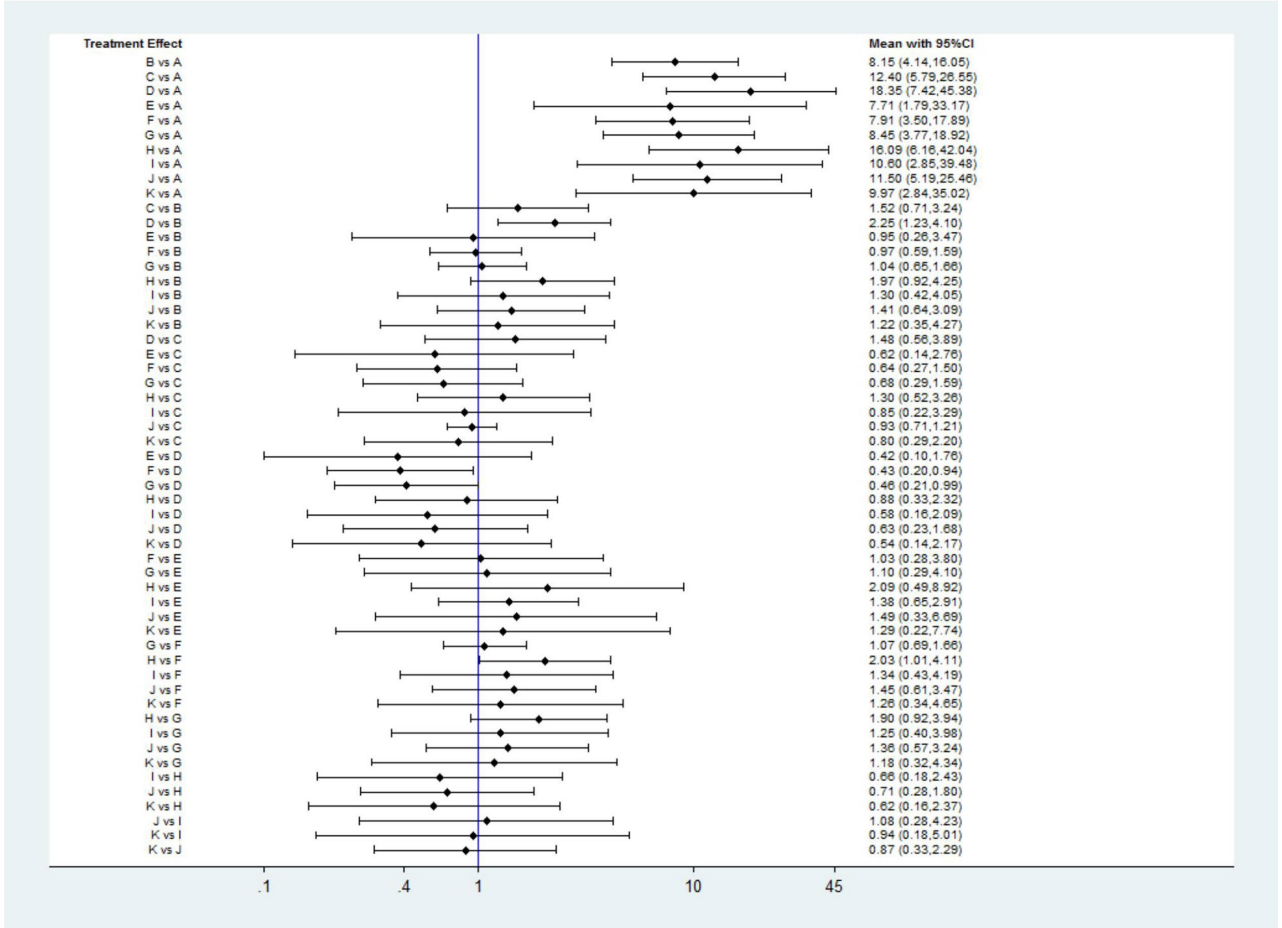
Forest plot of network meta-analysis for the 4-week ulcer healing rate in patients with peptic ulcer disease. A, Placebo; B, Omeprazole 20 mg; C, Lansoprazole 30 mg; D, Pantoprazole 40 mg; E, Rabeprazole 20 mg; F, Ilaprazole 5 mg; G, Ilaprazole 10 mg; H, Rabeprazole 10 mg; I, Ilaprazole 20 mg; J, Vonoprazan 20 mg; K, Esomeprazole 20 mg.

Twenty studies (14, 16–22, 28–33, 38, 42, 43, 45, 51, 52) that included eleven interventions reported the ulcer healing rate at 4 weeks. The network plot is shown in Figure 2B, and the results indicated that in terms of 4-week cure rate (Figure 4): PPIs and vonoprazan significantly improved the healing rates compared to placebo. The cure rate of pantoprazole 40 mg is higher than that of omeprazole 20 mg (OR = 2.25, 95%CI = 1.23–4.10) and rabeprazole 10 mg is superior to ilaprazole 5 mg (OR = 2.03, 95%CI = 1.01–4.11). Additionally, ilaprazole 5 mg (OR = 0.43, 95%CI = 0.20–0.94) and ilaprazole 10 mg (OR = 0.46, 95%CI = 0.21–0.99) are inferior to pantoprazole 40 mg in improving the ulcer healing rate. Table 2 and Supplementary Figure S2A present the ranking of all interventions based on the SUCRA: pantoprazole 40 mg ranked first, followed by rabeprazole 10 mg, lansoprazole 30 mg, vonoprazan 20 mg, ilaprazole 20 mg, esomeprazole 20 mg, ilaprazole 10 mg, rabeprazole 20 mg, omeprazole 20 mg, and ilaprazole 5 mg.

**Table 2:** Surface under the cumulative ranking score ranking for efficacy and safety outcomes of peptic ulcer disease.

Treatment		Efficacy outcomes						Safety outcomes	
		2 weeks	4 weeks				8 weeks	TEAEs	DRAEs
			PUD	PU	GU	DU			
Placebo	A	0.2	0.1	0.0	–	0.0	–	30.9	50.7
Omeprazole 20 mg	B	46.3	36.0	41.1	–	43.1	–	44.0	43.1
Lansoprazole 30 mg	C	78.2	67.8	74.6	69.1	78.3	87.4	77.3	76.0
Pantoprazole 40 mg	D	–	86.4	91.5	–	–	–	57.3	–
Rabeprazole 20 mg	E	54.1	38.5	–	–	–	–	68.9	–
Ilaprazole 5 mg	F	23.5	34.0	35.9	–	42.7	–	50.0	22.5
Ilaprazole 10 mg	G	73.4	39.4	43.3	–	62.0	–	68.5	59.7
Rabeprazole 10 mg	H	–	80.5	–	–	–	–	45.6	29.4
Ilaprazole 20 mg	I	–	57.0	–	–	–	–	–	–
Vonoprazan 20 mg	J	74.3	59.2	63.9	45.9	73.9	53.7	46.7	57.7
Esomeprazole 20 mg	K	–	51.1	–	35.8	–	8.9	19.1	44.3
Vonoprazan 10 mg	L	–	–	–	–	–	–	41.7	66.6

PUD, peptic ulcer disease; PU, peptic ulcer; GU, gastric ulcer; DU, duodenal ulcer; TEAEs, treatment-emerged adverse events; DRAEs, drug related adverse events.

**Figure 5:**
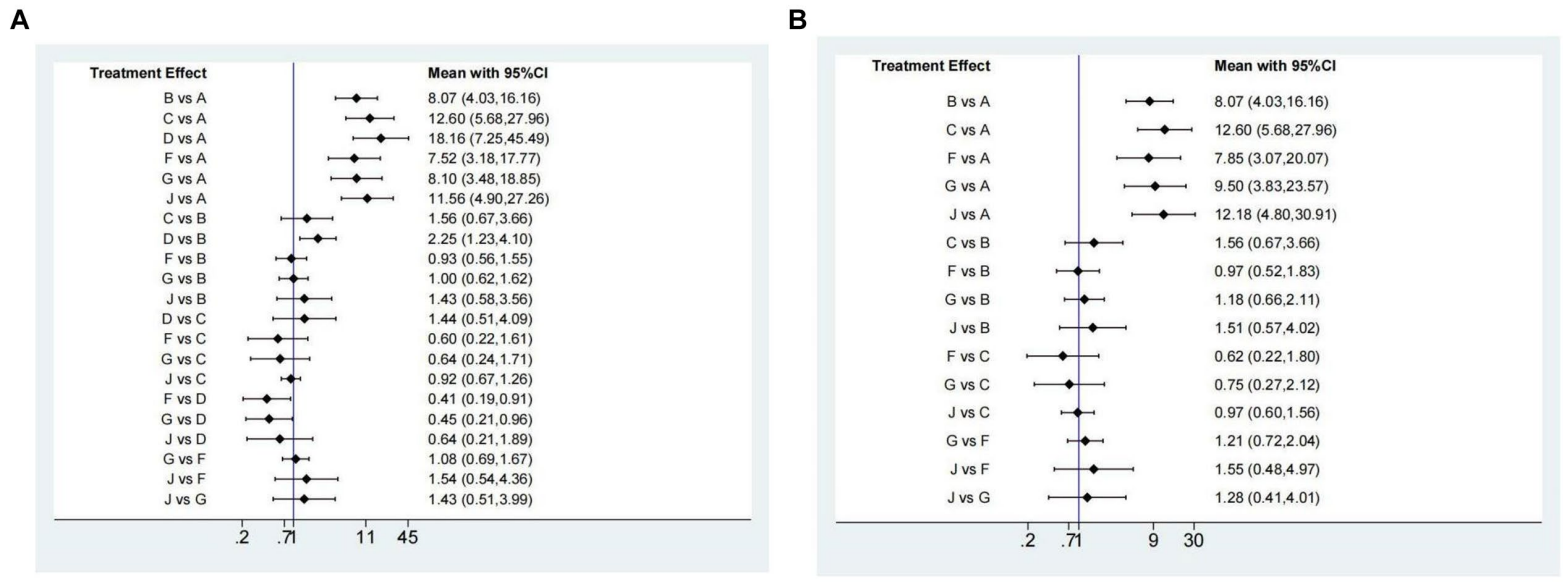
Forest plot of network meta-analysis for the 4-week ulcer healing rate in patients with peptic ulcer **(A)** and duodenal ulcer **(B)**. A, Placebo; B, Omeprazole 20 mg; C, Lansoprazole 30 mg; D, Pantoprazole 40 mg; F, Ilaprazole 5 mg; G, Ilaprazole 10 mg; J, Vonoprazan 20 mg.

In addition, we also analyzed ulcer healing rates for peptic ulcer at 4 weeks by excluding ESD-induced gastric ulcer. Twelve studies (14, 17, 28–33, 42, 43, 45, 52) have reported the ulcer healing rate at 4 weeks, and seven interventions were involved. The network plot is shown in Figure 2C. As shown in Figure 5A, the results showed that all the included interventions significantly improved the 4 weeks ulcer healing rate, compared with placebo. Furthermore, both ilaprazole 10 mg (OR = 0.45, 95% CI = 0.21-0.96) and ilaprazole 5 mg (OR = 0.41, 95% CI = 0.19-0.91) demonstrate inferior efficacy compared to pantoprazole 40 mg in the 4-week healing of peptic ulcers. Additionally, pantoprazole 40 mg exhibits superior efficacy relative to omeprazole 20 mg (OR = 2.25, 95% CI = 1.23-4.10). The SUCRA ranks of efficacy of all the included interventions is shown in Table 2 and Supplementary Figure S2B, pantoprazole 40 mg ranks first, sequentially followed by lansoprazole 30 mg, vonoprazan 20 mg, ilaprazole 10 mg, omeprazole 20 mg and ilaprazole 5 mg.

#### Ulcer healing rate at 8 weeks

Nine studies (14–20, 22, 53) that included three interventions have reported the ulcer healing rates at 8 weeks. As shown in Supplementary Figure S3A, there was no significant difference in the ulcer healing rate among all the included interventions. The results of SUCRA indicated that the relative ranking efficacy was: lansoprazole 30 mg, vonoprazan 20 mg and esomeprazole 20 mg (Table 2; Supplementary Figure S3B). However, there are insufficient data to analyze the effect of drugs on the efficacy of 8 weeks of peptic ulcer.

#### Healing rate of gastric ulcer and duodenal ulcer at 4 weeks

For gastric ulcer, six studies (14, 16, 18–20, 22) that included three interventions participated in the analysis. As presented in Supplementary Figure S3C, there was no significant difference in the ulcer healing among the included interventions. The SUCRA ranks of efficacy of all investigated drugs are shown in Table 2 and Supplementary Figure S3D. Lansoprazole 30 mg ranks first, sequentially followed by vonoprazan 20 mg and esomeprazole 20 mg.

Ten studies (14, 17, 28–31, 42, 43, 45, 52) have reported the ulcer healing rate for duodenal ulcer at 4 weeks, and five interventions were involved. As shown in Figure 5B, compared with placebo, all the included interventions significantly improved the 4 weeks ulcer healing rate. The SUCRA ranks of efficacy of all investigated drugs are shown in Table 2 and Supplementary Figure S4. Lansoprazole 30 mg ranks first, sequentially followed by vonoprazan 20 mg, ilaprazole 10 mg, omeprazole 20 mg and ilaprazole 5 mg.

#### Ulcer healing rate at 4 weeks and 8 weeks of ESD-induced gastric ulcer

Five studies (16, 18–20, 22) at 4 weeks and seven studies (15, 16, 18–20, 22, 53) at 8 weeks have reported the ulcer healing rate of ESD-induced gastric ulcer involving three interventions. According to the network meta-analysis results, there was no significant difference in the ulcer healing rate among all the above interventions at 4 weeks (Supplementary Figure S5A) and 8 weeks (Supplementary Figure S5B). The SUCRA results showed that lansoprazole 30 mg had the highest cumulative probability (4 weeks 64.1% vs. 8 weeks 80.5%), followed by vonoprazan 20 mg (4 weeks 49.3% vs. 8 weeks 60.8%), esomeprazole 20 mg (4 weeks 36.6% vs. 8 weeks 8.8%) (Supplementary Figures S5C,D).

### Safety outcomes

#### TEAEs

**Figure 6:**
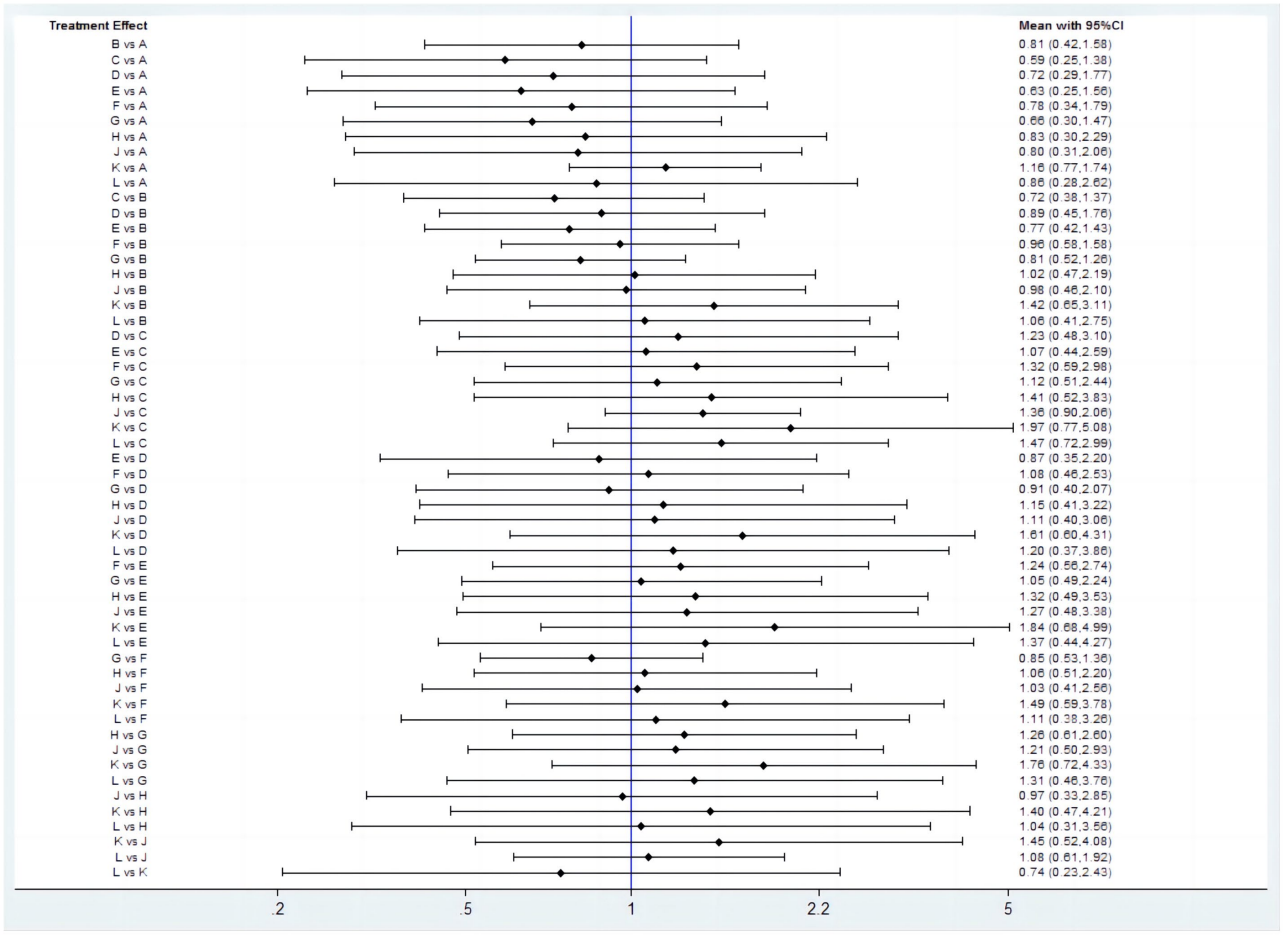
Forest plot of network meta-analysis for treatment-emergent adverse events. A, Placebo; B, Omeprazole 20 mg; C, Lansoprazole 30 mg; D, Pantoprazole 40 mg; E, Rabeprazole 20 mg; F, Ilaprazole 5 mg; G, Ilaprazole 10 mg; H, Rabeprazole 10 mg; J, Vonoprazan 20 mg; k, Esomeprazole 20 mg; L, Vonoprazan 10 mg.

Twenty-five studies (14, 17, 28–47, 49, 50, 52) encompassing eleven interventions reported data on TEAEs for PUD. The network plot is presented in Figure 2D. Among these studies, the five most frequently reported adverse events associated with various PPIs were headache, diarrhea, nausea, constipation, and abdominal pain. In contrast, the most common adverse events associated with vonoprazan group were diarrhea, constipation, nausea, infections, and fractures. No significant difference in the incidence of TEAEs was observed across all interventions (Figure 6). The results of SUCRA indicated that lansoprazole 30 mg ranks the first, followed by rabeprazole 20 mg, ilaprazole 10 mg, pantoprazole 40 mg, ilaprazole 5 mg, vonoprazan 20 mg, rabeprazole 10 mg, vonoprazan 10 mg, placebo and esomeprazole 20 mg (Table 2; Supplementary Figure S6A).

#### DRAEs

**Figure 7:**
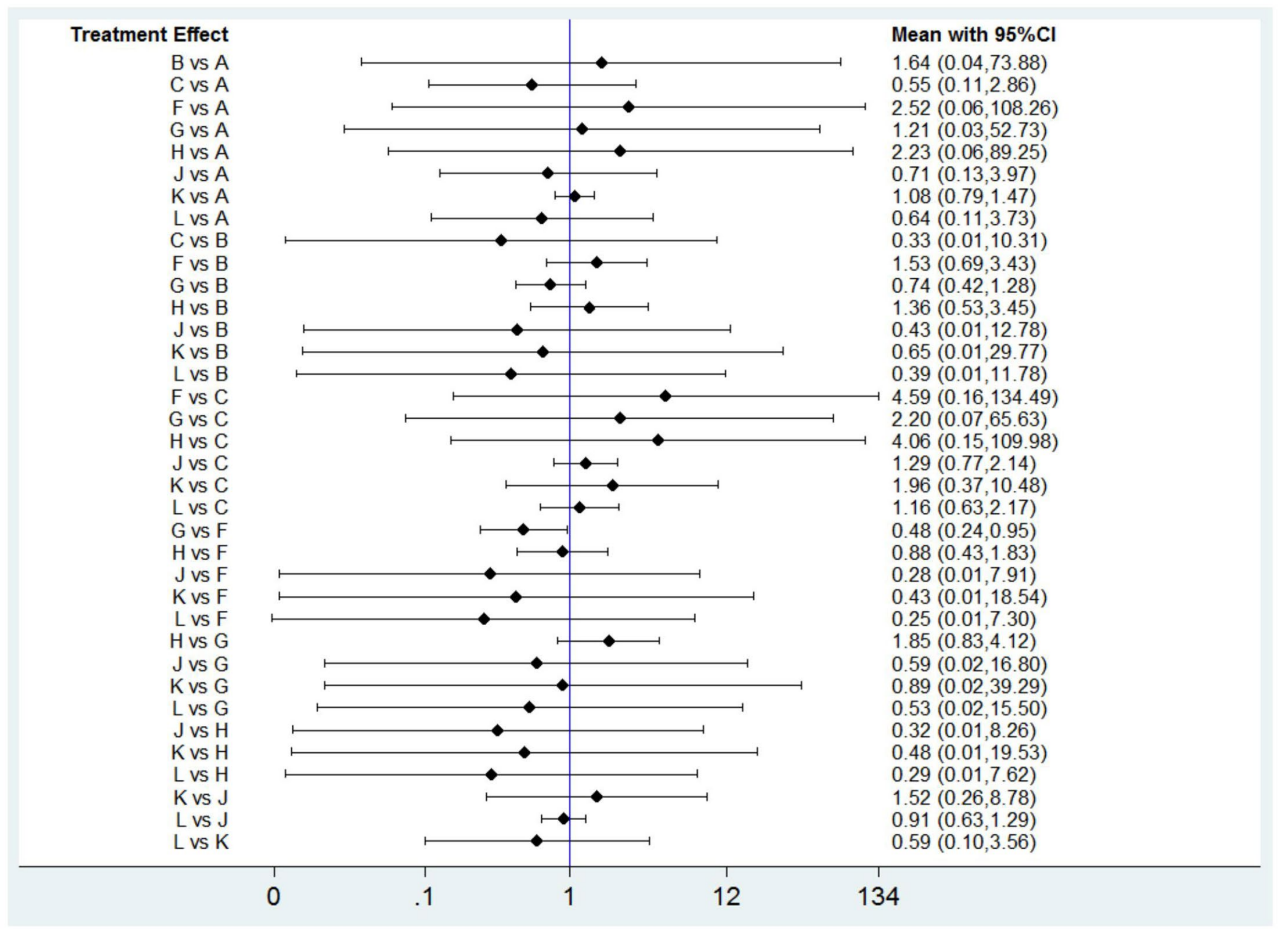
Forest plot of network meta-analysis for drug-related adverse events. A, Placebo; B, Omeprazole 20 mg; C, Lansoprazole 30 mg; F, Ilaprazole 5 mg; G, Ilaprazole 10 mg; H, Rabeprazole 10 mg; J, Vonoprazan 20 mg; K, Esomeprazole 20 mg; L, Vonoprazan 10 mg.

DRAEs were reported in eleven studies (14, 17, 35, 43–47, 49, 50, 52) involving nine interventions. There was no significant difference in the incidence of DRAEs among the majority of PPIs, vonoprazan, and placebo (Figure 7). However, the data indicated that ilaprazole 10 mg is associated with a lower incidence of DRAEs compared to ilaprazole 5 mg (OR=0.48, 95%CI=0.23-0.95).The results of SUCRA indicated that the relative ranking safety was: lansoprazole 30 mg ranked first, followed by vonoprazan 10 mg, ilaprazole 10 mg, vonoprazan 20 mg, placebo, esomeprazole 20 mg, omeprazole 20 mg, rabeprazole 10 mg and ilaprazole 5 mg (Table 2 and Supplementary Figure S6B).

### Complications

#### Delayed bleeding

Five studies (15, 16, 18, 20, 22) on ESD-induced gastric ulcer included 711 participants and the investigators reported delayed bleeding complications in patients treated with vonoprazan and PPIs. According to the results, there was no significant difference in the delayed bleeding rate among all the included interventions (Supplementary Figure S7A). The results of SUCRA indicated that the relative ranking safety was vonoprazan 20 mg, lansoprazole 30 mg and esomeprazole 20 mg (Supplementary Figure S7B).

#### Ulcer perforation

Six studies (15, 16, 18, 20, 22, 53) on ESD-induced gastric ulcer included 850 participants and the investigators reported ulcer perforation complications in patients treated with vonoprazan 20 mg, esomeprazole 20 mg, and lansoprazole 30 mg. There was no significant difference in the ulcer perforation rate among all the included interventions based on the network meta-analysis results (Supplementary Figure S7C). The results of SUCRA indicated that the relative ranking safety was lansoprazole 30 mg, vonoprazan 20 mg and esomeprazole 20 mg (Supplementary Figure S7D).

#### Evaluation of inconsistency

Results of evaluation of the inconsistency for all comparisons are presented in Supplementary Table S3 and Supplementary Figures S8, S9. We noted a significance level of *p* > 0.05 for all cases, which indicated that inconsistency was not present in any comparison. Thus, the consistency hypothesis was accepted in this study.

#### Publication bias

The visual examination of the funnel plots did not indicate the presence of publication bias for ulcer healing rates at 4 weeks and 8 weeks, TEAEs, and DRAEs in our network meta-analysis. Furthermore, Egger’s test results corroborated the absence of small-study effects. The funnel plots and corresponding *P*-values from Egger’s test are detailed in Supplementary Figure S10 and Supplementary Table S4.

## Discussion

PUD is a globally prevalent condition associated with considerable morbidity and mortality (1, 2). Acid suppression plays a pivotal role in the management of PUD (2). It is widely accepted that the enhancement of PUD treatment is achieved through the suppression of gastric acid secretion, with super efficacy attained when sustaining an intragastric pH > 3 for as long as possible with a 24-h period (54, 55). Although PPIs are currently the most commonly used acid suppressants, their effectiveness has been under scrutiny due to their inherent limited acid inhibition ability. Vonoprazan is a novel, potent, and highly selective P-CABs with an acting mechanism different from PPIs. Previous pharmacodynamic studies have affirmed that vonoprazan is more effective than lansoprazole in maintaining intragastric pH > 3 for this extended duration (56, 57). Another study reported that the pH 4 holding time ratios of vonoprazan were significantly longer than those of esomeprazole and rabeprazole (58). Vonoprazan was expected to exhibit more pronounced efficacy in acid-related diseases, including PUD.

We combined available direct and indirect evidence from 25 RCTs to evaluate the efficacy of different doses of vonoprazan and PPIs in the treatment of PUD. The results showed that all included anti-ulcer regimens had better efficacy than placebo, and the efficacy varied depending on the duration of treatment for PUD. Moreover, there was no significant difference between vonoprazan and all included PPIs. However, based on the SUCRA results, lansoprazole 30 mg ranked first in terms of the healing rate of 2 and 8 weeks and pantoprazole 40 mg ranked first in terms of the healing rate of 2 weeks. Additionally, lansoprazole 30 mg ranked first for safety in terms of TEAEs and DRAEs. From this point of view, lansoprazole 30 mg could be considered as the optimal treatment for PUD. Furthermore, subgroup analysis also confirmed the efficacy of pantoprazole 40 mg in the treatment of peptic ulcer and lansoprazole 30 mg in the treatment of gastric ulcer and duodenal ulcer at 4 weeks.

To date, only one network meta-analysis (59) has been performed to investigate the efficacy and safety of vonoprazan compared with PPIs in the treatment of peptic ulcer. This study confirmed the superiority of vonoprazan to PPIs in treating peptic ulcer, which included 45 direct and indirect comparisons. In addition, the study also showed that vonoprazan had a moderate risk of adverse events, and the rates of DRAEs was higher than those of lansoprazole, which was consistent with our study. However, our network meta-analysis did not substantiate the superiority of vonoprazan in patients with either PUD or peptic ulcer. It is critical to acknowledge that prior network meta-analyses arrived at their conclusions by incorporating data from varying treatment durations into a single quantitative synthesis. This methodological approach may have introduced bias and potentially influenced the outcomes. Treatment duration is a crucial factor in ulcer healing, with extended treatment periods often resulting in enhanced healing (14). The clinical significance of faster ulcer healing cannot be overlooked, as it can lead to quicker symptom relief, reduce the risk of complications, and shorten the overall treatment course, which is highly beneficial for patients. Our study addressed this by investigating the efficacy of all strategies separately according to different treatment durations, which significantly increased the reliability of the pooled results. Furthermore, the dosage of the drug represents a critical variable influencing ulcer healing. Previous study, however, did not account for the impact of different drug dosages on their findings, thereby introducing significant bias due to unit-of-analysis errors. Taking this critical factor into account, our study evaluated and ranked various doses of vonoprazan and PPIs based on their relative efficacy, providing clinicians with more practical recommendations for decision making, which is a notable strength of this study.

Recently, several meta-analyses have been conducted to evaluate the efficacy of vonoprazan and PPIs in the treatment of ESD-induced gastric ulcer. Some of the studies (18, 19) demonstrated the superiority of vonoprazan compared with PPIs, while other studies (60, 61) verified the non-inferiority of vonoprazan to PPIs in the ulcer healing of ESD-induced gastric ulcer. Chen et al. (62) conducted an updated meta-analysis directly compared the efficacy of vonoprazan with PPIs, and the results confirmed there were no significant differences in terms of ulcer healing, shrinkage rates, or ulcer perforation rates between vonoprazan and PPIs. One potential reason for this difference is the use of different PPIs in individual studies, and drug metabolism differs depending on the types of PPIs. Ulcer healing might be affected by different PPIs across studies. Thus, it remains unclear whether vonoprazan is superior to PPIs in the healing of ESD-induced gastric ulcer.

We conducted a subgroup analysis on ESD-induced gastric ulcer to compare the efficacy of vonoprazan with PPIs using direct and indirect evidence, and no significant difference was found between vonoprazan 20 mg, lansoprazole 30 mg, and esomeprazole 20 mg. Based on the SUCRA results, lansoprazole 30 mg ranked first for the ulcer healing rate at 4 and 8 weeks among lansoprazole 30 mg, esomeprazole 20 mg, and vonoprazan 20 mg in patients with ESD-induced gastric ulcer. Most studies administered injections of PPIs before ESD (15, 16, 18, 19, 48), while a few studies administered PPIs orally early (20, 53). This approach may be related to an increase in gastric pH value, partially helping to observe the rapidity and effectiveness of the healing process, and may lead to bias in overestimating the efficacy of PPIs. Moreover, PPIs are prodrugs, which have a gradual onset of action and typically reach a steady state of efficacy after 3–5 days of intake (9). Early administrations accelerate the onset time of PPIs, causing them to reach a steady state earlier, which may affect the results and introduce bias; hence, the results should be interpreted with caution.

As a newer drug, vonoprazan is relatively more expensive than traditional PPIs such as lansoprazole. According to the World Health Organization’s Model List of Essential Medicines, most of PPIs is listed as an essential medicine, while vonoprazan is not (63). Based on our findings, we conclude that lansoprazole 30 mg and pantoprazole 40 mg are not only more effective and safer than vonoprazan 20 mg, but also more cost-effective and accessible. Lansoprazole 30 mg may be the optimal regimen for treating PUD at 2 and 8 weeks, and pantoprazole 40 mg may be optimal regimen in treating PUD and peptic ulcer, especially in settings with limited healthcare resources. However, in groups or individuals at high risk for acid-suppression, vonoprazan could be more effective since it does not affect by the CYP2C19 polymorphism (12, 13).

However, there are still some limitations in this study. Firstly, a subgroup analysis predicated on *H. pylori* infection status was not executed, attributable to the insufficiency of pertinent data within this study. We acknowledge the significance of *H. pylori* status in comprehending the comprehensive efficacy of ulcer treatments and its prospective influence on treatment outcomes. We plan to update the results once we obtain the necessary data. Secondly, studies publish in language other than English were excluded, which could result in potential bias. Thirdly, vonoprazan was first approved in Japan, and most of the included studies were performed in Japan (14–16, 18–22, 48–50, 53), urgently requiring further researches from other countries to confirm our results. Fourthly, the number of interventions and studies included in each subgroup are inconsistent, and the results should be interpreted with caution. More head to head studies are needed to demonstrate the reliability of outcomes. Fifthly, we have included data on artificial gastric ulcers in the analysis of PUD. Although the treatment strategies for both artificial gastric ulcers and traditional peptic ulcers involve inhibiting acid secretion and promoting ulcer healing, the pathogenesis of peptic ulcers and artificial gastric ulcers is different, which may lead to some differences. More studies focusing on peptic ulcers are needed to clarify the efficacy of vonoprazan compared with various PPIs.

## Conclusion

The finding of this network meta-analysis suggested lansoprazole 30 mg may be the optimal regimen to increase the ulcer healing rate of 2 and 8 weeks of PUD, whereas pantoprazole 40 mg performed best in 4-week ulcer healing of PUD. Subgroup analysis showed that lansoprazole 30 mg was the most efficacious regimen for 4 weeks gastric ulcer and duodenal ulcer, while pantoprazole 40 mg was the optimal treatment in peptic ulcers. In terms of artificial gastric ulcer, lansoprazole 30 mg was the most effective regimen for ulcer healing at 4 and 8 weeks. Meanwhile, the safety of lansoprazole 30 mg might be superior to other treatment interventions in PUD. Our findings question the efficacy and safety of vonoprazan, and further direct head-to-head studies of vonoprazan and PPIs are needed to clarify the efficacy and safety of vonoprazan in clinical practice to guide clinical decision making and provide better treatment options.

## Data Availability

The original contributions presented in the study are included in the article/Supplementary material, further inquiries can be directed to the corresponding author.
