# Supplementary figures and images for: Efficacy and safety of vonoprazan versus proton pump inhibitors in the treatment of peptic ulcer disease: a systematic review and network meta-analysis for randomized controlled trails
Source: Front Nutr. 2024 Sep 5;11:1436993. doi: 10.3389/fnut.2024.1436993 (PMC11412081; doi:10.3389/fnut.2024.1436993)

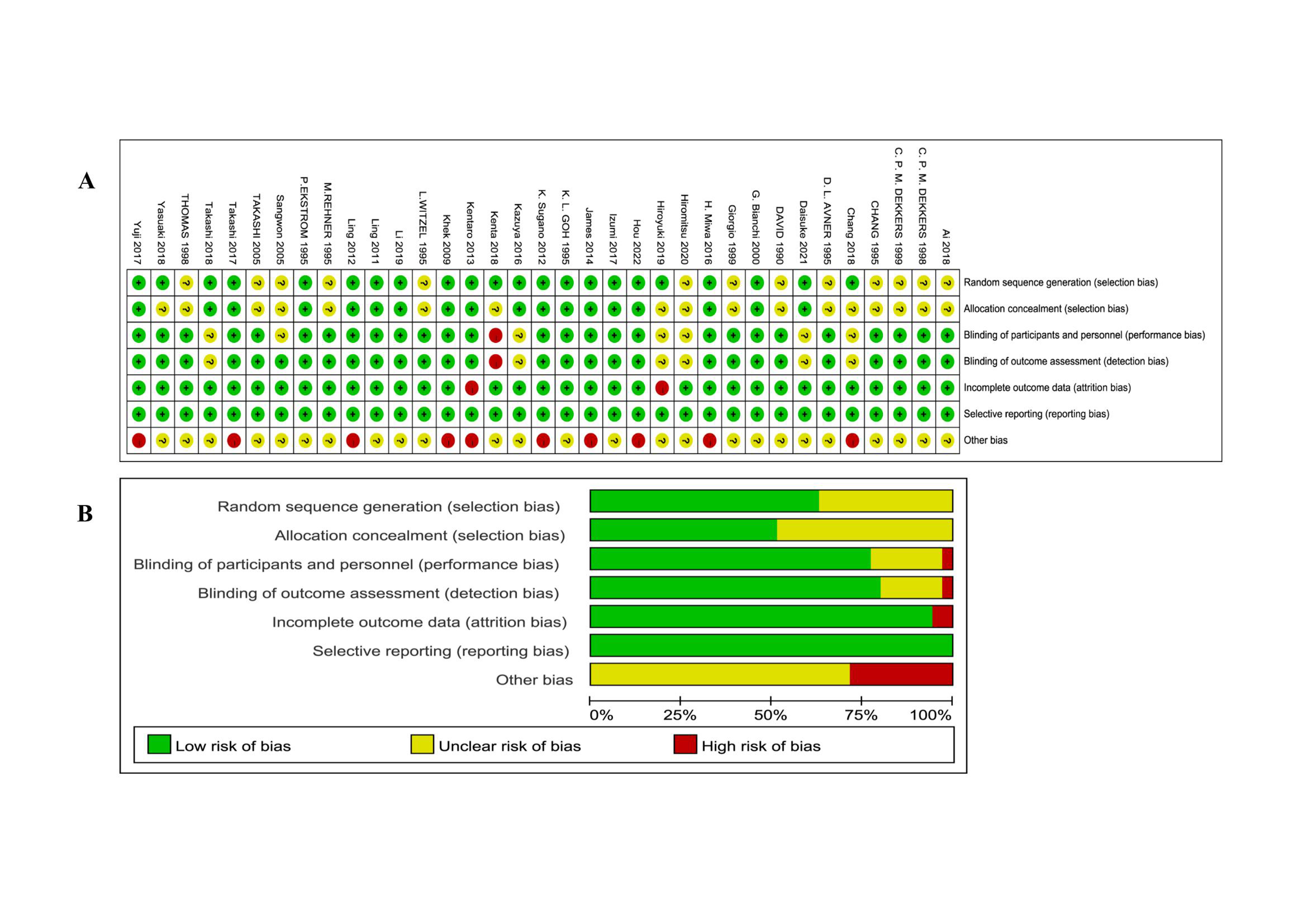

Supplement: Supplementary file 1 [file Image_1.JPEG]

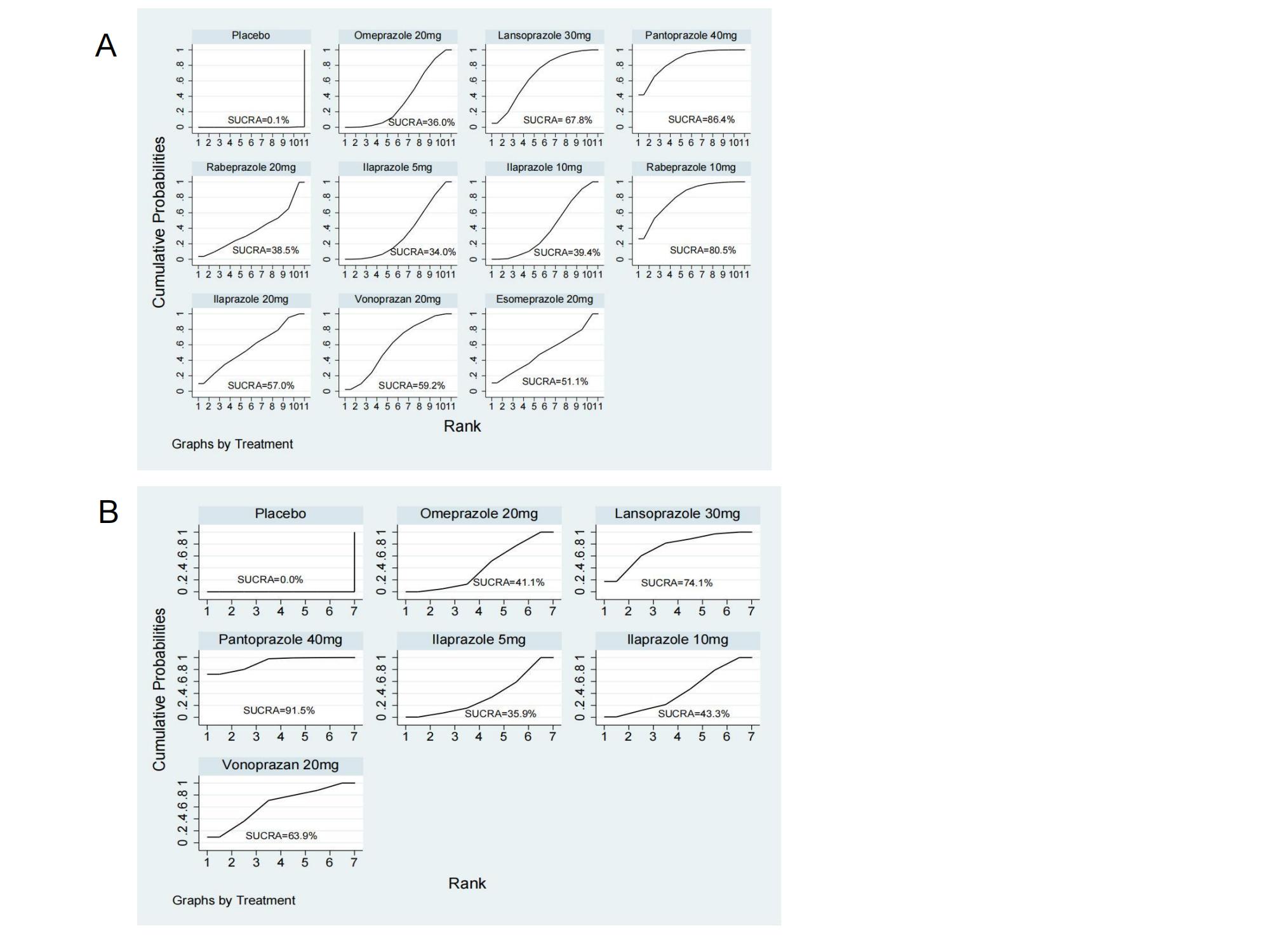

Supplement: Supplementary file 2 [file Image_2.JPEG]

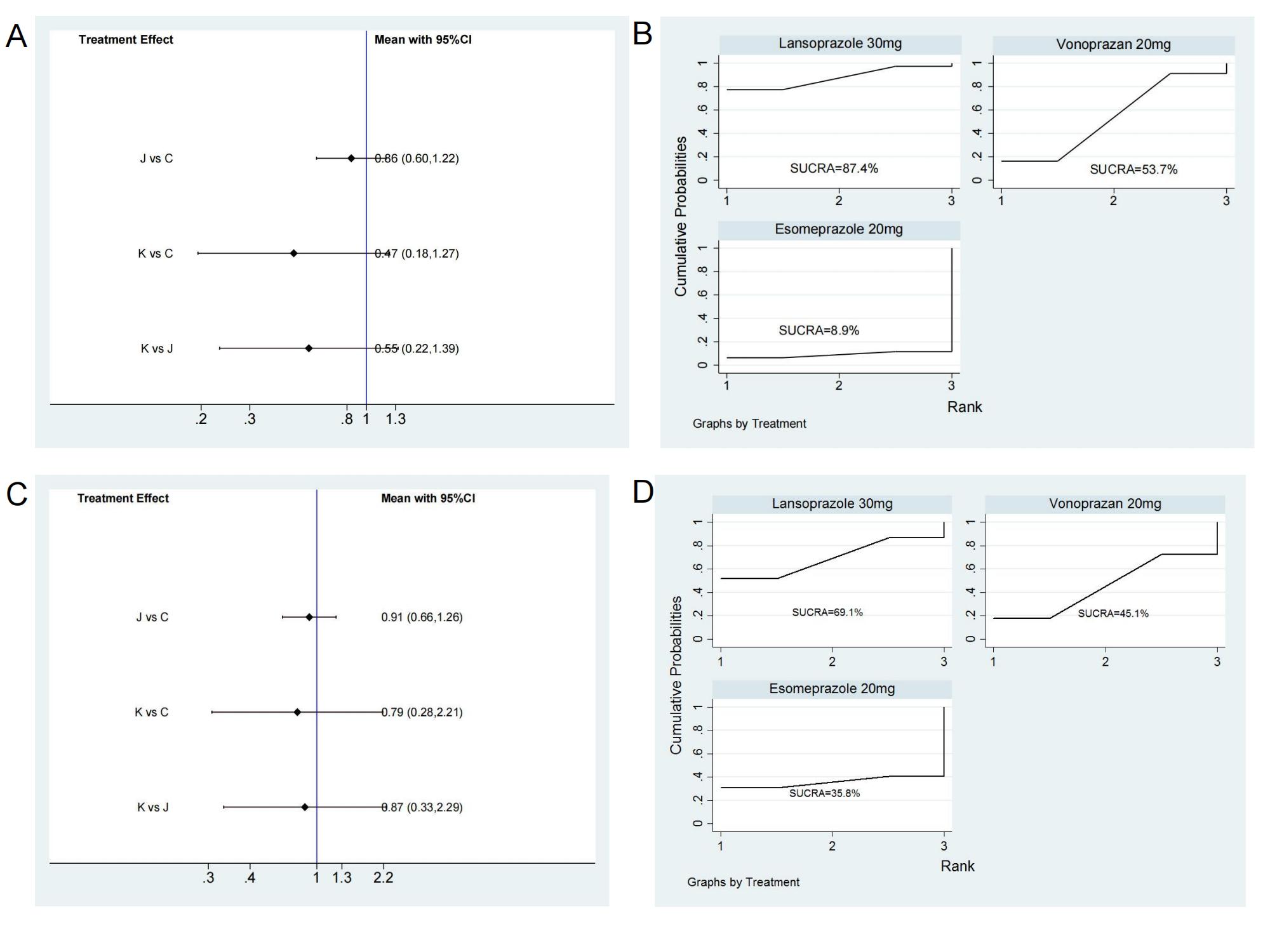

Supplement: Supplementary file 3 [file Image_3.JPEG]

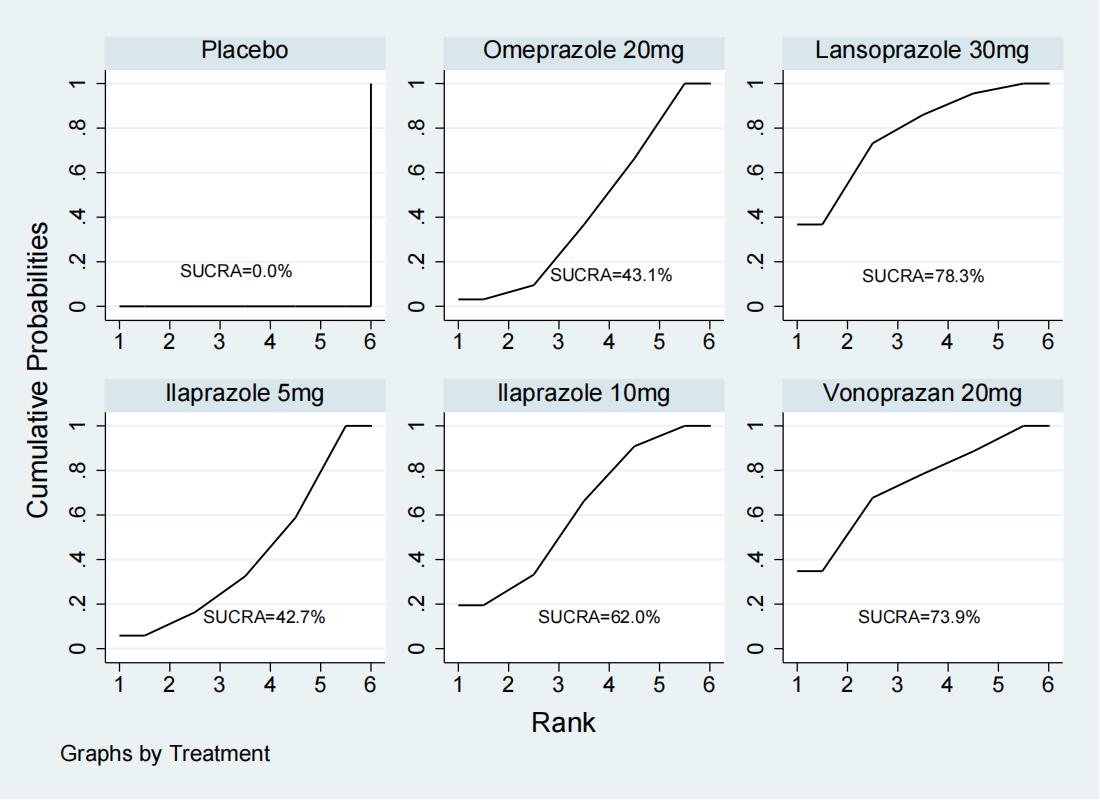

Supplement: Supplementary file 4 [file Image_4.JPEG]

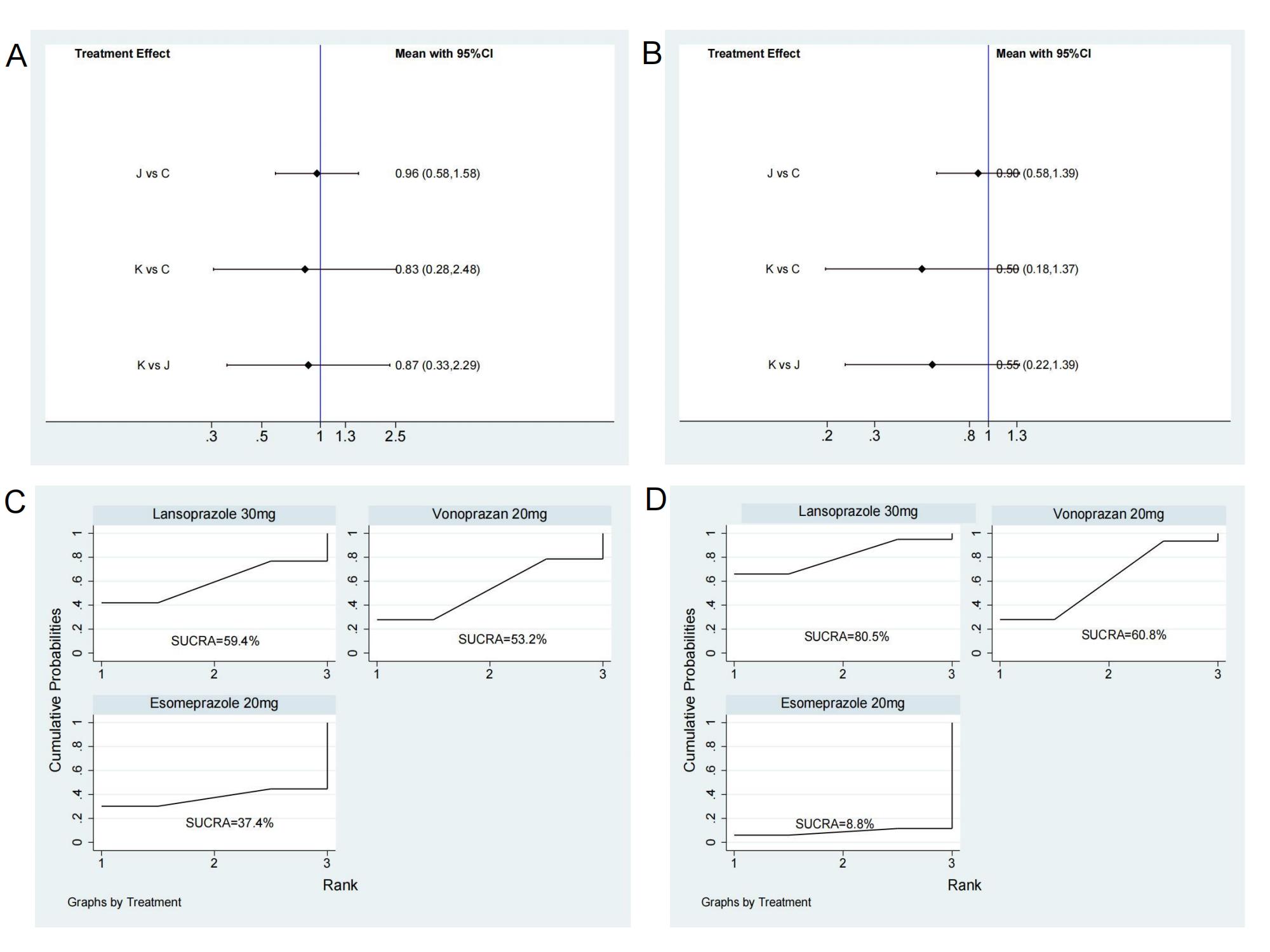

Supplement: Supplementary file 5 [file Image_5.JPEG]

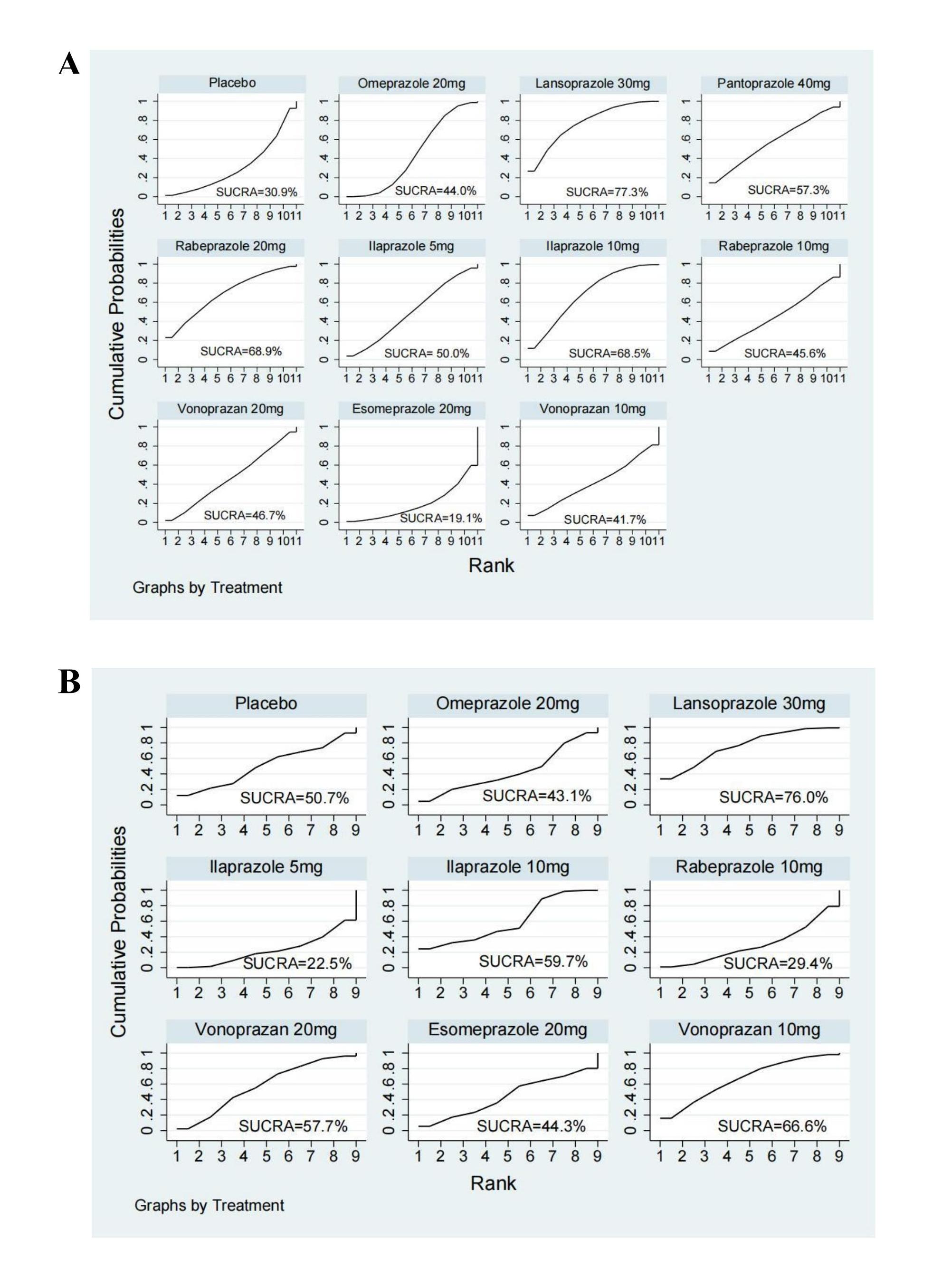

Supplement: Supplementary file 6 [file Image_6.jpg]

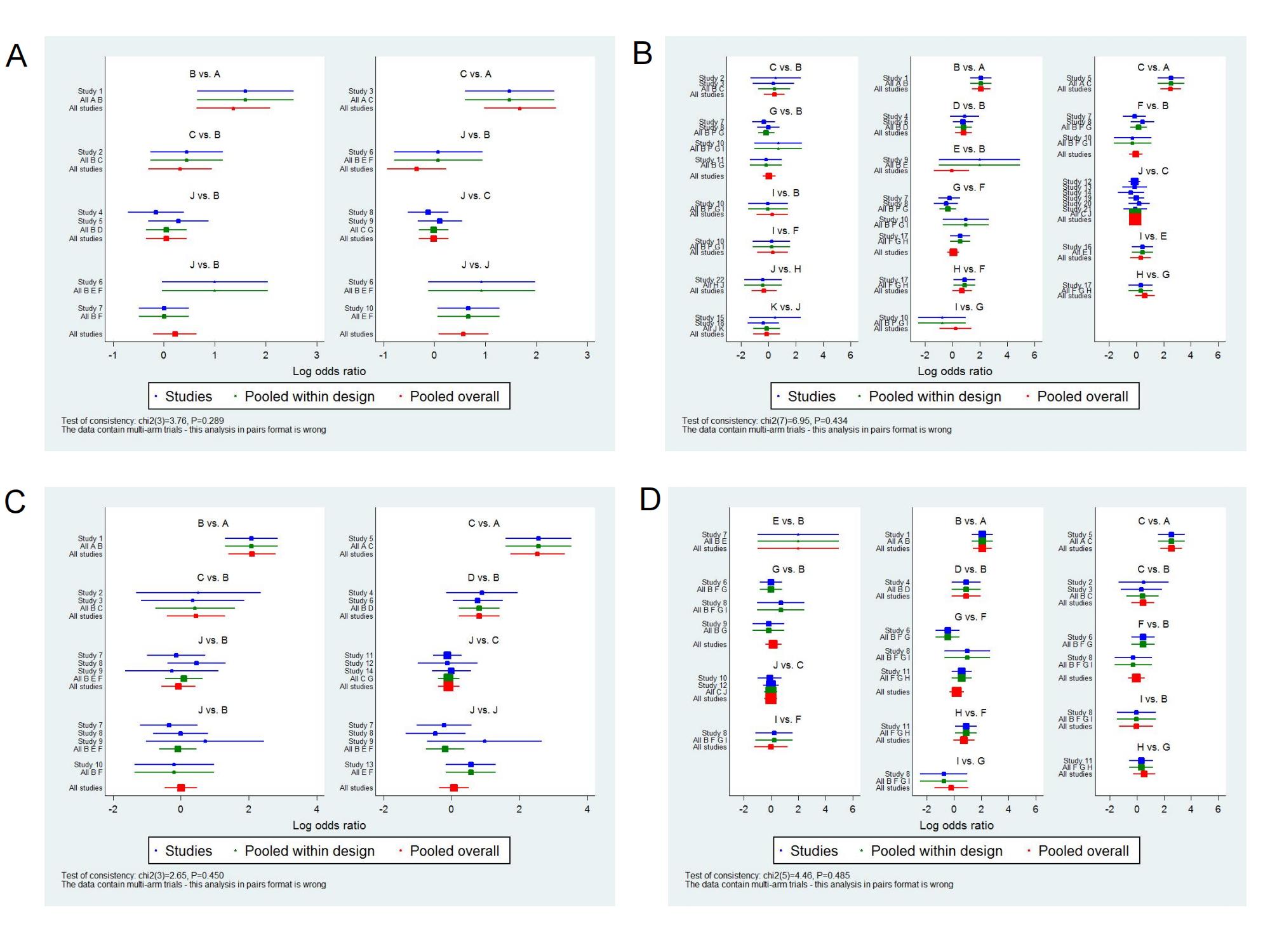

Supplement: Supplementary file 8 [file Image_8.JPEG]

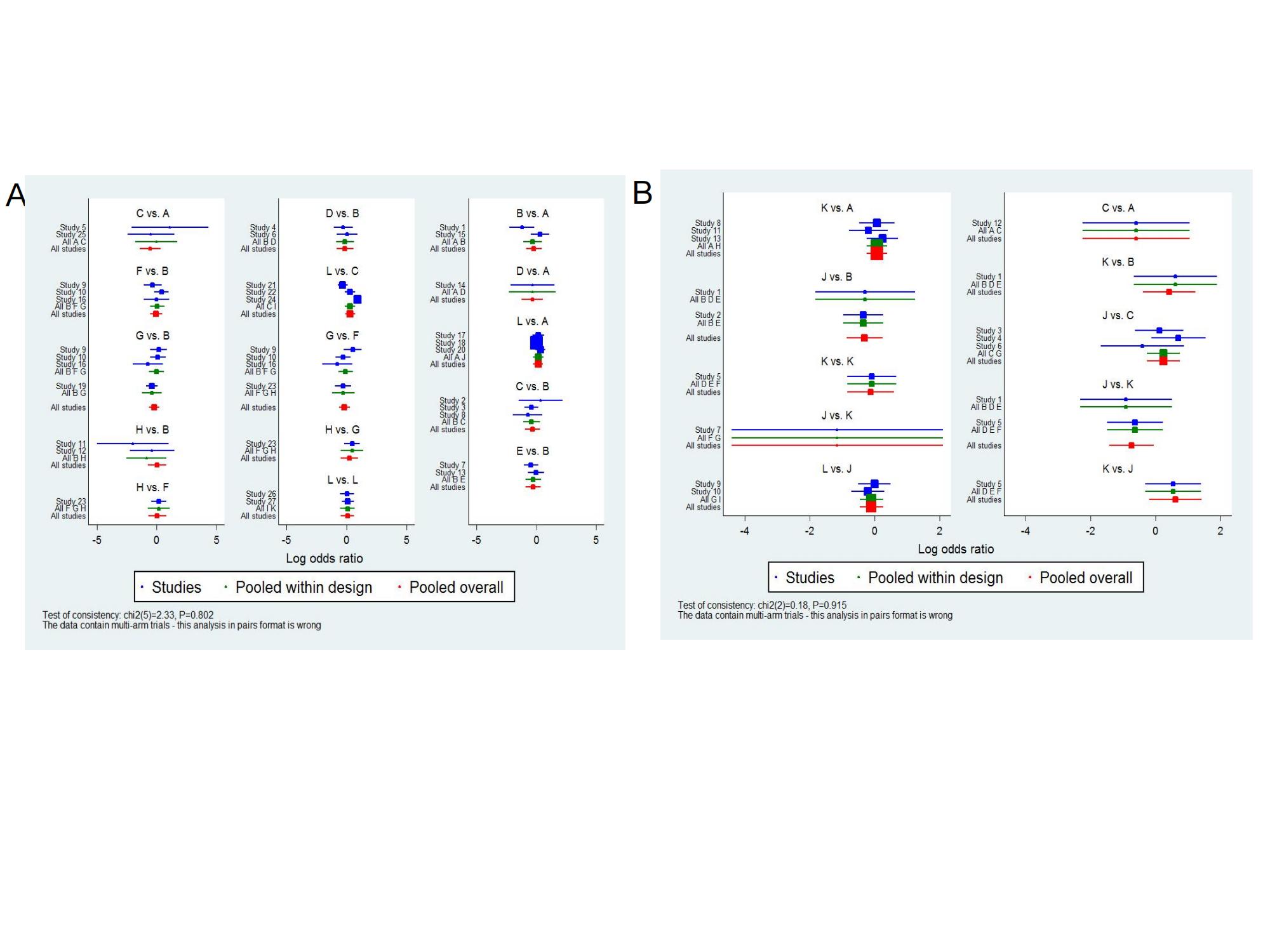

Supplement: Supplementary file 9 [file Image_9.JPEG]

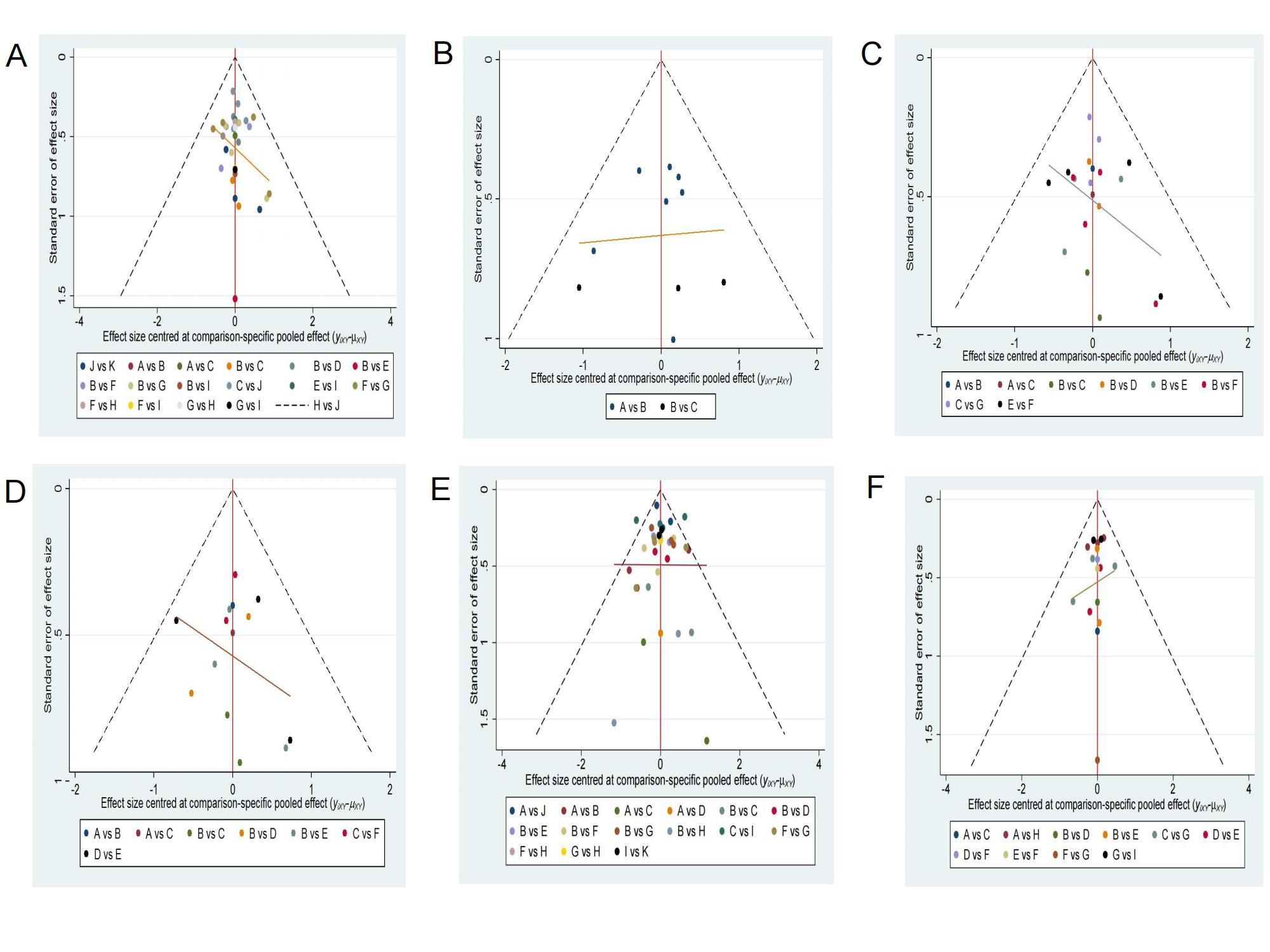

Supplement: Supplementary file 10 [file Image_10.JPEG]
