# Supplementary material for: Efficacy and safety of vonoprazan versus proton pump inhibitors in the treatment of peptic ulcer disease: a systematic review and network meta-analysis for randomized controlled trails
Source: Front Nutr. 2024 Sep 5;11:1436993. doi: 10.3389/fnut.2024.1436993 (PMC11412081; doi:10.3389/fnut.2024.1436993)
Supplement: Supplementary file 12 [file Table_2.docx]

**Supplementary Table S2** Search terms for this network meta-analysis.

| Search number | Query | Results |
| --- | --- | --- |
| 1 | "Peptic Ulcer"[Mesh] | 81,231 |
| 2 | Peptic Ulcer[Title/Abstract] OR Ulcer, Peptic[Title/Abstract] OR Ulcers, Peptic[Title/Abstract] OR Gastroduodenal Ulcer[Title/Abstract] OR Gastroduodenal Ulcers[Title/Abstract] OR Ulcer, Gastroduodenal[Title/Abstract] OR Ulcers, Gastroduodenal[Title/Abstract] OR Marginal Ulcer[Title/Abstract] OR Marginal Ulcers[Title/Abstract] OR Ulcer, Marginal[Title/Abstract] OR Ulcers, Marginal[Title/Abstract] | 35,489 |
| 3 | #1 OR #2 | 89,522 |
| 4 | "Stomach Ulcer"[Mesh] | 27,118 |
| 5 | Stomach Ulcer[Title/Abstract] OR Ulcer, Stomach[Title/Abstract] OR Ulcers, Stomach[Title/Abstract] OR Gastric Ulcer[Title/Abstract] OR Gastric Ulcers[Title/Abstract] OR Ulcer, Gastric[Title/Abstract] OR Ulcers, Gastric[Title/Abstract] | 17,042 |
| 6 | #4 OR #5 | 33,796 |
| 7 | "Duodenal Ulcer"[Mesh] | 25,512 |
| 8 | Duodenal Ulcer[Title/Abstract] OR Ulcer, Duodenal[Title/Abstract] OR Ulcers, Duodenal[Title/Abstract] OR Curling Ulcer[Title/Abstract] OR Ulcer, Curling[Title/Abstract] OR Curling's Ulcer[Title/Abstract] OR Curlings Ulcer[Title/Abstract] | 17,281 |
| 9 | #7 OR #8 | 29,981 |
| 10 | #3 OR #6 OR #9 | 96,164 |
| 11 | "1-(5-(2-fluorophenyl)-1-(pyridin-3-ylsulfonyl)-1H-pyrrol-3-yl)-N-methylmethanamine" [Supplementary Concept] | 353 |
| 12 | Vonoprazan[Title/Abstract] OR TAK 438[Title/Abstract] OR TAK438[Title/Abstract] OR TAK-438[Title/Abstract] OR potassium-competitive acid blocker[Title/Abstract] OR Vonaprazan[Title/Abstract] OR takecab[Title/Abstract] | 735 |
| 13 | #11OR #12 | 4,012,506 |
| 14 | "Proton Pump Inhibitors"[Mesh] | 13,939 |
| 15 | ((((Inhibitors, Proton Pump[Title/Abstract]) OR (Proton Pump Inhibitor[Title/Abstract])) OR (Inhibitor, Proton Pump[Title/Abstract])) OR (Pump Inhibitor, Proton[Title/Abstract])) OR (PPI[Title/Abstract]) | 37,073 |
| 16 | #14 OR #15 | 43,976 |
| 17 | "ilaprazole" [Supplementary Concept] | 56 |
| 18 | (((Ilaprazole[Title/Abstract]) OR (IY 81149[Title/Abstract])) OR (IY81149[Title/Abstract])) OR (IY-81149[Title/Abstract]) | 97 |
| 19 | #17 OR #18 | 100 |
| 20 | "Dexlansoprazole"[Mesh] | 86 |
| 21 | Dexlansoprazole[Title/Abstract] OR Lansoprazole, R-Isomer[Title/Abstract] OR Lansoprazole, R Isomer[Title/Abstract] OR R-Isomer Lansoprazole[Title/Abstract] OR 2-((R)-((3-Methyl-4-(2,2,2-trifluoroethoxy)-2-pyridinyl)methyl)sulfinyl)-1H-benzimidazole[Title/Abstract] OR R-Lansoprazole[Title/Abstract] OR R Lansoprazole[Title/Abstract] OR Dexlansoprazole Sesquihydrate[Title/Abstract] OR TAK 390MR[Title/Abstract] OR TAK390MR[Title/Abstract] OR TAK-390MR[Title/Abstract] OR TAK-390[Title/Abstract] OR TAK 390[Title/Abstract] OR TAK390[Title/Abstract] OR Dexilant[Title/Abstract] OR T-168390[Title/Abstract] OR T 168390[Title/Abstract] OR T168390[Title/Abstract] | 38 |
| 22 | #20 OR #21 | 105 |
| 23 | "Esomeprazole"[Mesh] | 1,279 |
| 24 | Esomeprazole Sodium[Title/Abstract] OR Esomeprazole Strontium[Title/Abstract] OR Strontium, Esomeprazole[Title/Abstract] OR Esomeprazole Magnesium[Title/Abstract] OR Nexium[Title/Abstract] OR Esomeprazole Potassium[Title/Abstract] OR Esomeprazole Strontium Anhydrous[Title/Abstract] | 263 |
| 25 | #23 OR #24 | 1,397 |
| 26 | "Lansoprazole"[Mesh] | 2,276 |
| 27 | Lansoprazol[Title/Abstract] OR 2-(((3-Methyl-4-(2,2,2-trifluoroethoxy)-2-pyridyl)methyl)sulfinyl)benzimidazole[Title/Abstract] OR Lansoprazoles[Title/Abstract] OR Ogastro[Title/Abstract] OR AG 1749[Title/Abstract] OR AG-1749[Title/Abstract] OR AG1749[Title/Abstract] OR Agopton[Title/Abstract] OR Bamalite[Title/Abstract] OR Lansol[Title/Abstract] OR Lansoprazole Sodium[Title/Abstract] OR Sodium, Lansoprazole[Title/Abstract] OR Lanzor[Title/Abstract] OR Monolitum[Title/Abstract] OR Opiren[Title/Abstract] OR Prevacid[Title/Abstract] OR Pro Ulco[Title/Abstract] OR Promeco[Title/Abstract] OR Takepron[Title/Abstract] OR Ulpax[Title/Abstract] OR Zoton[Title/Abstract] OR Ogast[Title/Abstract] OR Prezal[Title/Abstract] | 52 |
| 28 | #26 OR #27 | 2,286 |
| 29 | "Omeprazole"[Mesh] | 10,144 |
| 30 | Prilosec[Title/Abstract] OR Omeprazole Sodium[Title/Abstract] OR Sodium, Omeprazole[Title/Abstract] OR H 168-68[Title/Abstract] OR H 168 68[Title/Abstract] OR H 16868[Title/Abstract] OR Omeprazole Magnesium[Title/Abstract] OR Magnesium, Omeprazole[Title/Abstract] | 222 |
| 31 | #29 OR #30 | 10,222 |
| 32 | "Pantoprazole"[Mesh] | 1,480 |
| 33 | (((((((((SK[Title/Abstract] AND F 96022[Title/Abstract]) OR (SKF-96022[Title/Abstract])) OR (SKF 96022[Title/Abstract])) OR (SKF96022[Title/Abstract])) OR (SK[Title/Abstract] AND F-96022[Title/Abstract])) OR (SK[Title/Abstract] AND F96022[Title/Abstract])) OR (Protonix[Title/Abstract])) OR (BY 1023[Title/Abstract])) OR (BY-1023[Title/Abstract])) OR (Pantoprazole Sodium[Title/Abstract]) | 109 |
| 34 | #32 OR #33 | 1,525 |
| 35 | "Rabeprazole"[Mesh] | 1,081 |
| 36 | 2-((4-(3-methoxypropoxy)-3-methylpyridin-2-yl)methylsulfinyl)-1H-benzimidazole[Title/Abstract] OR E 3810[Title/Abstract] OR Dexrabeprazole[Title/Abstract] OR E3810[Title/Abstract] OR Pariet[Title/Abstract] OR Rabeprazole Sodium[Title/Abstract] OR Sodium, Rabeprazole[Title/Abstract] OR 1H-Benzimidazole, 2-(((4-(3-methoxypropoxy)-3-methyl-2-pyridinyl)methyl)sulfinyl)-, Sodium Salt[Title/Abstract] OR Aciphex[Title/Abstract] OR LY-307640[Title/Abstract] OR LY 307640[Title/Abstract] OR LY307640[Title/Abstract] | 16 |
| 37 | #35 OR #36 | 1,086 |
| 38 | #16 OR #19 OR #22 OR #25 OR #28 OR #31 OR #34 OR #37 | 51,266 |
| 39 | #10 AND #38 | 6,212 |
| 40 | (((((((randomized controlled trial[Publication Type]) OR (controlled clinical trial[Publication Type])) OR (randomized[Title/Abstract])) OR (randomised[Title/Abstract])) OR (placebo[Title/Abstract])) OR (randomly[Title/Abstract])) OR (random[Title/Abstract])) OR (trial[Title/Abstract]) | 2,114,670 |
| 41 | #39 AND #40 | 1,804 |
| 42 | #39 AND #40 Filters: English, Humans | 1,804 |
