# Supplementary material for: Efficacy and safety of vonoprazan versus proton pump inhibitors in the treatment of peptic ulcer disease: a systematic review and network meta-analysis for randomized controlled trails
Source: Front Nutr. 2024 Sep 5;11:1436993. doi: 10.3389/fnut.2024.1436993 (PMC11412081; doi:10.3389/fnut.2024.1436993)
Supplement: Supplementary file 13 [file Table_3.docx]

**Supplementary Table S3** Results of node-splitting models.

| **Outcomes** | **Comparison** | **Direct effect** | **Indirect effect** | **Overall** | **P-value** |
| --- | --- | --- | --- | --- | --- |
| 2 weeks ulcer healing of PUD | A vs B | 1.5990(0.4850) | 1.0218(0.5812) | 0.5812(0.5773) | 0.446 |
|  | A vs C | 1.4716(0.4514) | 2.0476(0.6077) | 0.6077(-0.5760) | 0.447 |
|  | B vs C | 0.4494(0.3663) | -0.1272(0.6625) | 0.6625(0.5765) | 0.446 |
|  | B vs D | 0.0484(0.2048) | -2.7264(93.01) | 93.0086(2.7748) | 0.976 |
|  | B vs E | 0.0804(0.4414) | -0.7038(0.3985) | 0.3985(0.7842) | 0.187 |
|  | B vs F | 0.1891(0.2671) | 0.7460(1.0372) | 1.0372(-0.5569) | 0.613 |
|  | C vs G | -0.0175(0.1495) | -3.3532(95.3735) | 95.3735(3.3357) | 0.972 |
|  | E vs F | 0.7320(0.2690) | -1.0107(0.9479) | 0.9479(1.7427) | 0.084 |
| 4 weeks ulcer healing of PUD | A vs B | 2.0756（0.3988) | 2.1621（0.6937) | -0.0865（0.8002) | 0.914 |
|  | A vs C | 2.552（0.4937) | 2.4655（0.6297) | 0.0866（0.8002) | 0.914 |
|  | B vs C | 0.4185（0.5968) | 0.4245（0.5074) | -0.006（0.7833) | 0.994 |
|  | B vs D | 0.8113（0.3065) | -4.3555（1637.254) | 5.1668（1637.254) | 0.997 |
|  | B vs E | 1.9865（1.5186) | -0.5368（0.7366) | 2.5233（1.6878) | 0.135 |
|  | B vs F | 0.0913（0.2816) | -0.5114（0.56) | 0.6027（0.6277) | 0.337 |
|  | B vs G | -0.1139（0.2549) | 1.1483（0.6976) | -1.2622（0.7431) | 0.089 |
|  | B vs I | -0.038（0.7338) | 0.7575（0.9422) | -0.7955（1.1939) | 0.505 |
|  | C vs J | -0.0851（0.1354) | -0.1983（0.9402) | 0.1132（0.9499) | 0.905 |
|  | E vs I | 0.4551（0.3928) | -2.0682（1.6415) | 2.5233（1.6878) | 0.135 |
|  | F vs G | 0.0828（0.2274) | -0.4611（1.2879) | 0.5439（1.3086) | 0.678 |
|  | F vs H | 0.9346（0.4036) | 0.0149（0.667) | 0.9196（0.7454) | 0.217 |
|  | F vs I | 0.2665（0.699) | 0.3471（0.9782) | -0.0806（1.1749) | 0.945 |
|  | G vs H | 0.3029（0.4423) | 1.3732（0.639) | -1.0703（0.762) | 0.160 |
|  | G vs I | -0.8494（0.8785) | 1.5097（0.9749) | -2.3591（1.4292) | 0.099 |
|  | H vs J | -0.4055（0.7071) | -0.2923（0.6343) | -0.1132（0.9499) | 0.905 |
|  | J vs K | -0.143（0.4967) | -4.831（1383.652) | 4.688（1383.652) | 0.997 |
| 4 weeks ulcer healing of PU | A vs B | 2.0756（0.3988) | 2.1335（0.7745) | -0.0579（0.8711) | 0.947 |
|  | A vs C | 2.552（0.4937) | 2.4941（0.7177) | 0.0579（0.8711) | 0.947 |
|  | B vs C | 0.4185（0.5968) | 0.4765（0.6346) | -0.0579（0.8711) | 0.947 |
|  | B vs D | 0.8113（0.3065) | -4.3389（1435.497) | 5.1502（1435.497) | 0.997 |
|  | B vs E | 0.089（0.2816) | -0.9491（0.6624) | 1.0381（0.7206) | 0.15 |
|  | B vs F | -0.1108（0.255) | 1.4068（0.9008) | -1.5176（0.938) | 0.106 |
|  | C vs G | -0.0858（0.1617) | -5.0554（1061.81) | 4.9696（1061.81) | 0.996 |
|  | E vs F | 0.0891（0.2275) | -0.3851（1.2945) | 0.4742（1.3144) | 0.718 |
| 4 weeks ulcer healing of DU | A vs B | 2.0756（0.3988) | 2.1329（0.7744) | -0.0573（0.8710) | 0.948 |
|  | A vs C | 2.5520（0.4937) | 2.4938（0.7177) | 0.0583（0.8711) | 0.947 |
|  | B vs C | 0.4185（0.5968) | 0.4765（0.6346) | -0.0579（0.8711) | 0.947 |
|  | B vs D | 0.2281（0.3695) | -0.8560（0.6730) | 1.0841（0.7727) | 0.161 |
|  | B vs E | 0.0123（0.3172) | 1.5217（1.0077) | -1.5094（1.0698) | 0.158 |
|  | C vs F | -0.0337（0.2457) | -5.0673（163.8288) | 5.0336（163.8290) | 0.975 |
|  | D vs E | 0.2280（0.2730) | -0.7211（1.3551) | 0.9491（1.3833) | 0.493 |
| TEAEs | A vs B | -0.3017(0.3901) | 0.1052(0.7067) | -0.4070(0.8073) | 0.614 |
|  | A vs C | -0.0366(0.8855) | -0.6898(0.5027) | 0.6532(1.0184) | 0.521 |
|  | A vs D | -0.3335(0.9882) | -0.3268(0.5191) | -0.0067(1.1162) | 0.995 |
|  | B vs C | -0.3993(0.3461) | 0.2539(0.9578) | -0.6532(1.0181) | 0.521 |
|  | B vs D | -0.1205(0.3742) | -0.1272(1.0517) | 0.0067(1.1162) | 0.995 |
|  | B vs E | -0.2580(0.3152) | 0.4034(105.392) | -0.6614(105.3924) | 0.995 |
|  | B vs F | 0.0571(0.2937) | -0.4114(0.5503) | 0.4685(0.6230) | 0.452 |
|  | B vs G | -0.1433(0.2401) | -0.9355(0.7865) | 0.7922(0.8236) | 0.336 |
|  | B vs H | -0.8470(0.8352) | 0.2554(0.4391) | -1.1024(0.9436) | 0.243 |
|  | C vs I | 0.3062(0.2118) | 0.7236(49.0427) | -0.4174(49.0432) | 0.993 |
|  | F vs G | -0.1294(0.2527) | -0.6497(0.9457) | 0.5202(0.9822) | 0.596 |
|  | F vs H | 0.1937(0.4488) | -0.2711(0.7039) | 0.4648(0.8379) | 0.579 |
|  | G vs H | 0.4800(0.4577) | -0.3123(0.6733) | 0.7924(0.8237) | 0.336 |
|  | I vs K | 0.0780(0.2938) | 0.4503(127.1344) | -0.3723(127.1347) | 0.998 |
| Drug-related AEs | A vs C | -0.5978(0.8412) | -0.5477(37.1017) | -0.0501(37.1112) | 0.999 |
|  | B vs D | 0.6268(0.6519) | 0.2935(0.5339) | 0.3333(0.8481) | 0.694 |
|  | B vs E | -0.3349(0.2915) | 0.2730(1.4215) | -0.6079(1.4589) | 0.677 |
|  | C vs G | 0.2522(0.2593) | 0.2560(40.1134) | -0.0037(40.1142) | 1.000 |
|  | D vs E | -0.7012(0.3728) | -1.0873(1.3890) | 0.3861(1.4734) | 0.793 |
|  | D vs F | -0.0818(0.3826) | -0.6893(1.4106) | 0.6076(1.4588) | 0.677 |
|  | E vs F | 0.5444(0.4411) | 1.1523(1.3583) | -0.6079(1.4587) | 0.677 |
|  | F vs G | -1.1486(1.6633) | -0.6194(61.8256) | -0.5292(61.8489) | 0.993 |
|  | G vs I | -0.0998(0.1820) | 0.6920(116.8044) | -0.7918(116.8045) | 0.995 |

Note: Data are Coefficient (Standard Error) in the column of “Direct effect”, “Indirect effect” and “Overall”.

Abbreviations: PUD, peptic ulcer disease; PU, peptic ulcer; DU, duodenal ulcer; TEAEs, treatment ermergent adverse events; AEs, adverse events; A, Placebo; B, Omeprazole 20mg; C, Lansoprazole 30mg; D, Pantoprazole 40mg; E, Rabeprazole 20mg; F, Ilaprazole 5mg; G, Ilaprazole 10mg; H, Rabeprazole 10mg; I, Ilaprazole 20mg; J, Vonoprazan 20mg; K, Esomeprazole 20mg; L, Vonoprazan 10mg.
