# Supplementary material for: Efficacy and safety of vonoprazan versus proton pump inhibitors in the treatment of peptic ulcer disease: a systematic review and network meta-analysis for randomized controlled trails
Source: Front Nutr. 2024 Sep 5;11:1436993. doi: 10.3389/fnut.2024.1436993 (PMC11412081; doi:10.3389/fnut.2024.1436993)
Supplement: Supplementary file 14 [file Table_4.docx]

**Supplementary Table S4** Evaluations for the potential bias within the network meta-analysis

| Groups of outcomes | | N | Egger's test (P value) | Publication bias |
| --- | --- | --- | --- | --- |
|  |  |  |  |  |
| Efficacy outcomes | |  |  |  |
| PUD | 4 weeks | 23 | 0.710 | No significant |
|  | 8 weeks | 10 | 0.288 | No significant |
| PU | 4 weeks | 14 | 0.332 | No significant |
| DU | 4 weeks | 10 | 0.843 | No significant |
| Safety outcomes | |  |  |  |
| TEAEs | | 27 | 0.106 | No significant |
| DRAEs | | 13 | 0.366 | No significant |

PUD, peptic ulcer disease; PU, peptic ulcer; DU, duodenal ulcer; GU, gastric ulcer; TEAEs, Treatment emergent adverse events; DRAEs, drug related adverse events.
